# Brain aging in major depressive disorder: results from the ENIGMA major depressive disorder working group

**DOI:** 10.1038/s41380-020-0754-0

**Published:** 2020-05-18

**Authors:** Laura K. M. Han, Richard Dinga, Tim Hahn, Christopher R. K. Ching, Lisa T. Eyler, Lyubomir Aftanas, Moji Aghajani, André Aleman, Bernhard T. Baune, Klaus Berger, Ivan Brak, Geraldo Busatto Filho, Angela Carballedo, Colm G. Connolly, Baptiste Couvy-Duchesne, Kathryn R. Cullen, Udo Dannlowski, Christopher G. Davey, Danai Dima, Fabio L. S. Duran, Verena Enneking, Elena Filimonova, Stefan Frenzel, Thomas Frodl, Cynthia H. Y. Fu, Beata R. Godlewska, Ian H. Gotlib, Hans J. Grabe, Nynke A. Groenewold, Dominik Grotegerd, Oliver Gruber, Geoffrey B. Hall, Ben J. Harrison, Sean N. Hatton, Marco Hermesdorf, Ian B. Hickie, Tiffany C. Ho, Norbert Hosten, Andreas Jansen, Claas Kähler, Tilo Kircher, Bonnie Klimes-Dougan, Bernd Krämer, Axel Krug, Jim Lagopoulos, Ramona Leenings, Frank P. MacMaster, Glenda MacQueen, Andrew McIntosh, Quinn McLellan, Katie L. McMahon, Sarah E. Medland, Bryon A. Mueller, Benson Mwangi, Evgeny Osipov, Maria J. Portella, Elena Pozzi, Liesbeth Reneman, Jonathan Repple, Pedro G. P. Rosa, Matthew D. Sacchet, Philipp G. Sämann, Knut Schnell, Anouk Schrantee, Egle Simulionyte, Jair C. Soares, Jens Sommer, Dan J. Stein, Olaf Steinsträter, Lachlan T. Strike, Sophia I. Thomopoulos, Marie-José van Tol, Ilya M. Veer, Robert R. J. M. Vermeiren, Henrik Walter, Nic J. A. van der Wee, Steven J. A. van der Werff, Heather Whalley, Nils R. Winter, Katharina Wittfeld, Margaret J. Wright, Mon-Ju Wu, Henry Völzke, Tony T. Yang, Vasileios Zannias, Greig I. de Zubicaray, Giovana B. Zunta-Soares, Christoph Abé, Martin Alda, Ole A. Andreassen, Erlend Bøen, Caterina M. Bonnin, Erick J. Canales-Rodriguez, Dara Cannon, Xavier Caseras, Tiffany M. Chaim-Avancini, Torbjørn Elvsåshagen, Pauline Favre, Sonya F. Foley, Janice M. Fullerton, Jose M. Goikolea, Bartholomeus C. M. Haarman, Tomas Hajek, Chantal Henry, Josselin Houenou, Fleur M. Howells, Martin Ingvar, Rayus Kuplicki, Beny Lafer, Mikael Landén, Rodrigo Machado-Vieira, Ulrik F. Malt, Colm McDonald, Philip B. Mitchell, Leila Nabulsi, Maria Concepcion Garcia Otaduy, Bronwyn J. Overs, Mircea Polosan, Edith Pomarol-Clotet, Joaquim Radua, Maria M. Rive, Gloria Roberts, Henricus G. Ruhe, Raymond Salvador, Salvador Sarró, Theodore D. Satterthwaite, Jonathan Savitz, Aart H. Schene, Peter R. Schofield, Mauricio H. Serpa, Kang Sim, Marcio Gerhardt Soeiro-de-Souza, Ashley N. Sutherland, Henk S. Temmingh, Garrett M. Timmons, Anne Uhlmann, Eduard Vieta, Daniel H. Wolf, Marcus V. Zanetti, Neda Jahanshad, Paul M. Thompson, Dick J. Veltman, Brenda W. J. H. Penninx, Andre F. Marquand, James H. Cole, Lianne Schmaal

**Affiliations:** 1 Department of Psychiatry, Amsterdam Public Health and Amsterdam Neuroscience, Amsterdam UMC, Vrije Universiteit & GGZinGeest, Amsterdam, The Netherlands; 2 Donders Institute for Brain, Cognition and Behavior, Radboud University, Nijmegen, The Netherlands; 3 Department of Psychiatry, University of Münster, Münster, Germany; 4 Imaging Genetics Center, Mark & Mary Stevens Neuroimaging & Informatics Institute, Keck School of Medicine, University of Southern California, Los Angeles, CA USA; 5 Desert-Pacific Mental Illness Research Education and Clinical Center, VA San Diego Healthcare, San Diego, CA USA; 6 Department of Psychiatry, University of California San Diego, Los Angeles, CA USA; 7 FSSBI “Scientific Research Institute of Physiology & Basic Medicine”, Laboratory of Affective, Cognitive & Translational Neuroscience, Novosibirsk, Russia; 8 Department of Neuroscience, Novosibirsk State University, Novosibirsk, Russia; 9 Department of Neuroscience, University Medical Center Groningen, University of Groningen, Groningen, The Netherlands; 10 Department of Clinical and Developmental Neuropsychology, University of Groningen, Groningen, The Netherlands; 11 Department of Psychiatry, Melbourne Medical School, The University of Melbourne, Melbourne, VIC Australia; 12 The Florey Institute of Neuroscience and Mental Health, The University of Melbourne, Melbourne, VIC Australia; 13 Institute of Epidemiology and Social Medicine, University of Münster, Münster, Germany; 14 Laboratory of Experimental & Translational Neuroscience, Novosibirsk State University, Novosibirsk, Russia; 15 Laboratory of Psychiatric Neuroimaging (LIM-21), Instituto de Psiquiatria, Hospital das Clinicas HCFMUSP, Faculdade de Medicina, Universidade de Sao Paulo, Sao Paulo, SP Brazil; 16 Department for Psychiatry, Trinity College Dublin, Dublin, Ireland; 17 North Dublin Mental Health Services, Dublin, Ireland; 18 Department of Biomedical Sciences, Florida State University, Tallahassee, FL USA; 19 Institute for Molecular Bioscience, University of Queensland, Brisbane, QLD Australia; 20 Department of Psychiatry and Behavioral Sciences, University of Minnesota Medical School, Minneapolis, Minnesota USA; 21 Orygen, The National Centre of Excellence in Youth Mental Health, Parkville, VIC Australia; 22 Centre for Youth Mental Health, The University of Melbourne, Melbourne, VIC Australia; 23 Department of Psychology, School of Arts and Social Sciences, City, University of London, London, UK; 24 Department of Neuroimaging, Institute of Psychiatry, Psychology & Neuroscience, King’s College, London, UK; 25 Department of Psychiatry and Psychotherapy, University Medicine Greifswald, Greifswald, Germany; 26 Department of Psychiatry and Psychotherapy, Otto von Guericke University (OVGU), Magdeburg, Germany; 27 German Center for Neurodegenerative Diseases (DZNE), Göttingen, Germany; 28 Centre for Affective Disorders, Institute of Psychiatry, Psychology & Neuroscience, King’s College London, London, UK; 29 School of Psychology, University of East London, London, UK; 30 Department of Psychiatry, University of Oxford, Oxford, UK; 31 Department of Psychology, Stanford University, Stanford, CA USA; 32 German Center of Neurodegenerative Diseases (DZNE) Site Rostock/Greifswald, Greifswald, Germany; 33 Interdisciplinary Center Psychopathology and Emotion regulation (ICPE), University Medical Center Groningen, University of Groningen, Groningen, The Netherlands; 34 Department of Psychiatry and Neuroscience Institute, University of Cape Town, Cape Town, South Africa; 35 Section for Experimental Psychopathology and Neuroimaging, Department of Psychiatry, University of Heidelberg, Heidelberg, Germany; 36 Department of Psychology, Neuroscience & Behaviour, McMaster University, Hamilton, ON Canada; 37 Melbourne Neuropsychiatry Centre, Department of Psychiatry, The University of Melbourne & Melbourne Health, Melbourne, VIC Australia; 38 Youth Mental Health Team, Brain and Mind Centre, University of Sydney, Sydney, NSW Australia; 39 Department of Neuroscience, University of California San Diego, San Diego, CA USA; 40 Department of Psychiatry & Behavioral Sciences, Standord University, Stanford, CA USA; 41 Department of Diagnostic Radiology and Neuroradiology, University Medicine Greifswald, Greifswald, Germany; 42 Department of Psychiatry, Philipps-University Marburg, Marburg, Germany; 43 Department of Psychology, University of Minnesota, Minneapolis, MN USA; 44 Department of Psychiatry and Psychotherapy, University of Bonn, Bonn, Germany; 45 Sunshine Coast Mind and Neuroscience Institute, University of the Sunshine Coast QLD, Sippy Downs, QLD Australia; 46 Departments of Psychiatry and Pediatrics, University of Calgary, Calgary, AB Canada; 47 Addictions and Mental Health Strategic Clinical Network, Calgary, AB Canada; 48 Department of Psychiatry, University of Calgary, Calgary, AB Canada; 49 Division of Psychiatry, University of Edinburgh, Edinburgh, UK; 50 Faculty of Medicine and Dentistry, University of Alberta, Edmonton, Alberta Canada; 51 School of Clinical Sciences, Queensland University of Technology, Brisbane, QLD Australia; 52 Institute of Health and Biomedical Innovation, Queensland University of Technology, Brisbane, QLD Australia; 53 QIMR Berghofer Medical Research Instititute, Brisbane, QLD Australia; 54 Department of Psychiatry and Behavioral Sciences, The University of Texas Health Science Center at Houston, Houston, TX USA; 55 Institut d’Investigació Biomèdica Sant Pau, Barcelona, Catalonia Spain; 56 Centro de Investigación Biomédica en Red de Salud Mental, Cibersam, Spain; 57 Department of Radiology and Nuclear Medicine, Amsterdam University Medical Centers, AMC, Amsterdam, The Netherlands; 58 Center for Depression, Anxiety, and Stress Research, McLean Hospital, Harvard Medical School, Belmont, MA USA; 59 Max Planck Institute of Psychiatry, Munich, Germany; 60 Department of Psychiatry and Psychotherapy, University Medical Center Göttingen, Göttingen, Germany; 61 Department of Psychiatry and Psychotherapy, Asklepios Fachklinikum Göttingen, Göttingen, Germany; 62 SA MRC Unit on Risk and Resilience, University of Cape Town, Cape Town, South Africa; 63 Queensland Brain Institute, University of Queensland, Brisbane, QLD Australia; 64 Cognitive Neuroscience Center, University Medical Center Groningen, University of Groningen, Groningen, The Netherlands; 65 Division of Mind and Brain Research, Department of Psychiatry and Psychotherapy CCM, Charité—Universitätsmedizin Berlin, corporate member of Freie Universität Berlin, Humboldt-Universität zu Berlin, and Berlin Institute of Health, Berlin, Germany; 66 Department of Child Psychiatry, University Medical Center, Leiden, The Netherlands; 67 Leiden Institute for Brain and Cognition, Leiden University, Leiden, The Netherlands; 68 Department of Psychiatry, Leiden University Medical Center, Leiden, The Netherlands; 69 Centre for Advanced Imaging, University of Queensland, Brisbane, QLD Australia; 70 Institute for Community Medicine, University Medicine Greifswald, Greifswald, Germany; 71 Department of Psychiatry, Division of Child and Adolescent Psychiatry, UCSF School of Medicine, UCSF, San Francisco, CA USA; 72 Faculty of Health, Queensland University of Technology, Brisbane, QLD Australia; 73 Department of Clinical Neuroscience, Osher Center, Karolinska Institutet, Stockholm, Sweden; 74 Department of Psychiatry, Dalhousie University, Halifax, NS Canada; 75 NORMENT Centre, Institute of Clinical Medicine, University of Oslo, Oslo, Norway; 76 Division of Mental Health and Addiction, Oslo University Hospital, Oslo, Norway; 77 Clinic for Mental Health and Dependency, C-L psychiatry and Psychosomatic Unit, Oslo University Hospital, Oslo, Norway; 78 Hospital Clinic, University of Barcelona, IDIBAPS, CIBERSAM, Barcelona, Catalonia Spain; 79 FIDMAG Germanes Hospitalàries Research Foundation, CIBERSAM, Barcelona, Catalonia Spain; 80 Centre for Neuroimaging & Cognitive Genomics (NICOG), Clinical Neuroimaging Laboratory, NCBES Galway Neuroscience Centre, College of Medicine Nursing and Health Sciences, National University of Ireland Galway, H91 TK33 Galway, Ireland; 81 MRC Centre for Neuropsychiatric Genetics and Genomics, Cardiff University, Cardiff, UK; 82 Norwegian Centre for Mental Disorders Research, Institute of Clinical Medicine, University of Oslo, Oslo, Norway; 83 Department of Neurology, Oslo University Hospital, Oslo, Norway; 84 UNIACT, Psychiatry Team, Neurospin, Atomic Energy Commission, Gif-Sur-Yvette, France; 85 Translational Psychiatry Team, Pôle de psychiatrie, Faculté de Médecine, APHP, Hôpitaux Universitaires Mondor, INSERM, U955, Créteil, France; 86 Cardiff University Brain Research Imaging Centre, Cardiff University, Cardiff, UK; 87 Neuroscience Research Australia, Randwick, Sydney, NSW Australia; 88 School of Medical Sciences, University of New South Wales, Sydney, NSW Australia; 89 Department of Psychiatry, University Medical Center Groningen, University of Groningen, Groningen, The Netherlands; 90 Université de Paris, Service Hospitalo-Universitaire, GHU Paris Psychiatrie & Neuroscience, F-75014 Paris, France; 91 Neuroscience Institute, University of Cape Town, Cape Town, South Africa; 92 Laureate Institute for Brain Research, Tulsa, OK USA; 93 Department of Psychiatry, School of Medicine, University of Sao Paulo (FMUSP), Sao Paulo, Brazil; 94 Department of Psychiatry and Neurochemistry, Institute of Neuroscience and Physiology, The Sahlgrenska Academy at the University of Gothenburg, Gothenburg, Sweden; 95 Department of Medical Epidemiology and Biostatistics, Karolinska Institutet, Stockholm, Sweden; 96 Department of Clinical Neuroscience, University of Oslo, Oslo, Norway; 97 Clinic for Psychiatry and Dependency, C-L psychiatry and Psychosomatic Unit, Oslo University Hospital, Oslo, Norway; 98 School of Psychiatry, University of New South Wales, Kingsford, Sydney, NSW Australia; 99 Black Dog Institute, Prince of Wales Hospital, Randwick, Sydney, NSW Australia; 100 Instituto de Radiologia, Hospital das Clinicas HCFMUSP, Faculdade de Medicina, Universidade de Sao Paulo, Sao Paulo, SP Brazil; 101 Department of Psychiatry and Neurology, CHU Grenoble Alpes, Université Grenoble Alpes, F-38000 Grenoble, France; 102 Inserm 1216, Grenoble Institut des Neurosciences, GIN, F-38000 Grenoble, France; 103 Department of Psychiatry, Amsterdam University Medical Centers, AMC, Amsterdam, The Netherlands; 104 Department of Psychiatry, Radboud University Medical Center, Nijmegen, The Netherlands; 105 Department of Psychiatry, University of Pennsylvannia Perelman School of Medicine, Philadelphia, PA USA; 106 Oxley College of Health Sciences, The University of Tulsa, Tulsa, OK USA; 107 West Region and Research Division, Institute of Mental Health, Singapore, Singapore; 108 Yong Loo Lin School of Medicine, National University of Singapore, Singapore, Singapore; 109 Valkenberg Psychiatric Hospital, Cape Town, South Africa; 110 Instituto de Ensino e Pesquisa, Hospital Sírio-Libanês, Sao Paulo, SP Brazil; 111 Department of Cognitive Neuroscience, Radboud University Medical Centre, Nijmegen, The Netherlands; 112 Centre for Medical Image Computing, Department of Computer Science, University College London, London, UK; 113 Dementia Research Centre, Institute of Neurology, University College London, London, UK

**Keywords:** Neuroscience, Depression

## Abstract

Major depressive disorder (MDD) is associated with an increased risk of brain atrophy, aging-related diseases, and mortality. We examined potential advanced brain aging in adult MDD patients, and whether this process is associated with clinical characteristics in a large multicenter international dataset. We performed a mega-analysis by pooling brain measures derived from T1-weighted MRI scans from 19 samples worldwide. Healthy brain aging was estimated by predicting chronological age (18–75 years) from 7 subcortical volumes, 34 cortical thickness and 34 surface area, lateral ventricles and total intracranial volume measures separately in 952 male and 1236 female controls from the ENIGMA MDD working group. The learned model coefficients were applied to 927 male controls and 986 depressed males, and 1199 female controls and 1689 depressed females to obtain independent unbiased brain-based age predictions. The difference between predicted “brain age” and chronological age was calculated to indicate brain-predicted age difference (brain-PAD). On average, MDD patients showed a higher brain-PAD of +1.08 (SE 0.22) years (Cohen’s *d* = 0.14, 95% CI: 0.08–0.20) compared with controls. However, this difference did not seem to be driven by specific clinical characteristics (recurrent status, remission status, antidepressant medication use, age of onset, or symptom severity). This highly powered collaborative effort showed subtle patterns of age-related structural brain abnormalities in MDD. Substantial within-group variance and overlap between groups were observed. Longitudinal studies of MDD and somatic health outcomes are needed to further assess the clinical value of these brain-PAD estimates.

## Introduction

Major depressive disorder (MDD) is associated with an increased risk of cognitive decline [1], metabolic dysregulation [2], and cellular aging [3, 4], indicating that the burden of MDD goes beyond mental ill-health and functional impairment, and extends to poor somatic health [5], and age-related diseases [6]. Moreover, MDD increases the risk of mortality [7], and not only through death by suicide [8]. Simultaneously, depression and aging have been linked to poor quality of life and increased costs for society and healthcare [9]. This underscores the importance of identifying brain aging patterns in MDD patients to determine whether and how they deviate from healthy patterns of aging.

Current multivariate pattern methods can predict chronological age from biological data (see Jylhava et al. [10] for a review) with high accuracy. Similarly, chronological age can be predicted from brain images, resulting in an estimate known as “brain age” [11]. Importantly, by calculating the difference between a person’s estimated brain age and their chronological age, one can translate a complex aging pattern across the brain into a single outcome: brain-predicted age difference (brain-PAD). A positive brain-PAD represents having an “older” brain than expected for a person of their chronological age, whereas a negative brain-PAD signals a “younger” brain than expected at the given chronological age. Higher brain-PAD scores have been associated with greater cognitive impairment, increased morbidity, and exposure to cumulative negative fateful life events [11, 12]. For a review summarizing brain age studies from the past decade, see Franke and Gaser [13].

Prior studies from the Enhancing NeuroImaging Genetics through Meta-analysis (ENIGMA)-MDD consortium with sample sizes over 9000 participants have shown subtle reductions in subcortical structure volumes in major depression that were robustly detected across many samples worldwide. Specifically, smaller hippocampal volumes were found in individuals with earlier age of onset and recurrent episode status [14]. In addition, different patterns of cortical alterations were found in adolescents vs. adults with MDD, suggesting that MDD may affect brain morphology (or vice versa) in a way that depends on the developmental stage of the individual [15]. Thus, subtle structural brain abnormalities have been identified in MDD. However, whether a diagnosis of MDD is associated with the multivariate metric of brain aging in a large dataset, and which clinical characteristics further impact this metric, remains elusive.

Accumulating evidence from studies suggests that, at the group level, MDD patients follow advanced aging trajectories, as their functional (e.g., walking speed, handgrip strength) [16] and biological state (e.g. telomeres, epigenetics, mitochondria) [17–20] reflects what is normally expected at an older age (i.e., biological age “outpaces” chronological age) [21]. It is important to examine whether biological aging findings in depression can be confirmed in a large heterogeneous dataset consisting of many independent samples worldwide, based on commonly derived gray matter measures. Only a handful of studies have investigated brain-PAD in people with psychiatric disorders, showing older brain-PAD in schizophrenia (SCZ), borderline personality disorder, and first-episode and at-risk mental state for psychosis, yet findings were less consistent in bipolar disorder (BD) (for an overview, see Cole et al. [22]).

Only three studies to date specifically investigated machine-learning-based brain aging in MDD—using relatively small samples of <211 patients, with inconsistent findings of a brain-PAD of +4.0 years vs. no significant differences [23–25]. The current study is the first to examine brain aging in over 6900 individuals from the ENIGMA MDD consortium (19 cohorts, 8 countries worldwide), covering almost the entire adult lifespan (18–75 years). Our additional aim was to build a new multisite brain age model based on FreeSurfer regions of interest (ROIs) that generalizes well to independent data to promote brain age model deployability and shareability. We hypothesized higher brain-PAD in MDD patients compared with controls. We also conducted exploratory analyses to investigate whether higher brain-PAD in MDD patients was associated with demographics (age, sex) and clinical characteristics such as disease recurrence, antidepressant use, remission status, depression severity, and age of onset of depression.

## Methods

### Samples

Nineteen cohorts from the ENIGMA MDD working group with data from MDD patients and controls (18–75 years of age) participated in this study. MDD was ascertained using the clinician-rated the 17-item Hamilton Depression Rating Scale (HDRS-17) in one cohort and diagnostic interviews in all other cohorts. Details regarding demographics, clinical characteristics, and exclusion criteria for each cohort may be found in Supplementary Tables S1–3. Because the literature suggests differential brain developmental trajectories by sex [26], we estimated brain age models separately for males and females. Sites with less than ten healthy controls were excluded from the training dataset and subsequent analyses (for exclusions see Supplementary Material). In total, we included data from *N* = 6989 participants, including *N* = 4314 controls (*N* = 1879 males; *N* = 2435 females) and *N* = 2675 individuals with MDD (*N* = 986 males; *N* = 1689 females). All sites obtained approval from the appropriate local institutional review boards and ethics committees, and all study participants or their parents/guardians provided written informed consent.

### Training and test samples

**Fig. 1:**
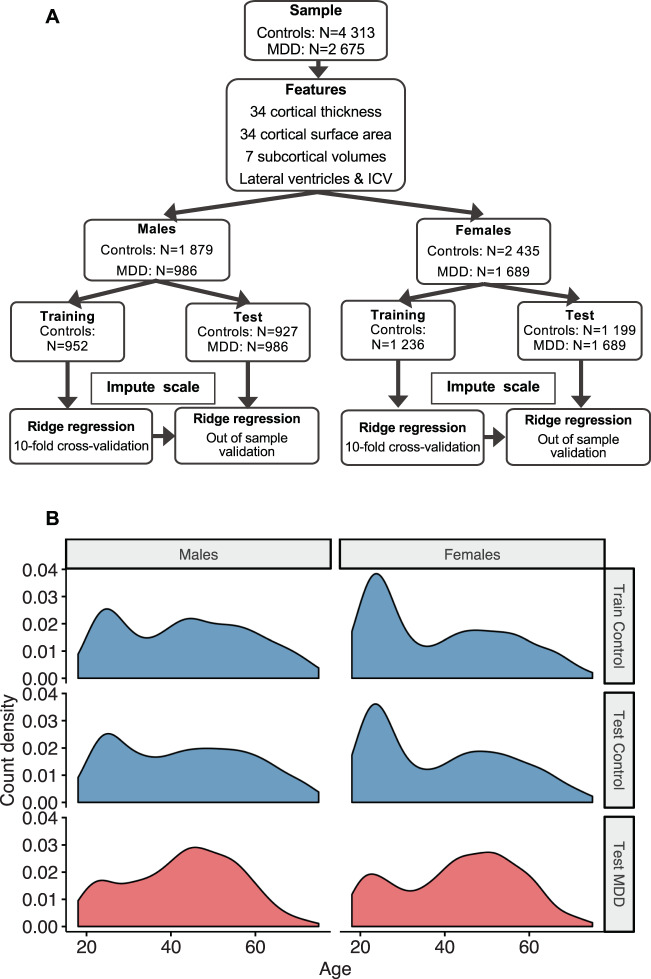
Data partition approach. **a** Schematic illustration of features used and data partition into training and test samples, separately for males and females. A full list of features can be found in the Supplementary Material. **b** Data from control groups (blue) were partitioned into balanced 50:50 splits within each scanning site following random sampling but preserving the overall chronological age distribution. Major depressive disorder (MDD) groups are shown in red. The top panel illustrates the male (left) and female (right) training samples. The middle and bottom panels show the male (controls: mean [SD] in years, 43.1 [15.3]; MDD: 42.8 [13.1]) and female test samples (controls: 39.4 [15.7]; MDD: 43.2 [14.0]). ICV intracranial volume.

To maximize the variation of chronological age distribution and scanning sites in the training samples, and to maximize the statistical power and sample size of patients for subsequent statistical analyses, we created balanced data splits within scanning sites preserving the chronological age distribution, Fig. 1a. The full motivation to our data partition approach can be found in the Supplementary Material. Structural brain measures from 952 males obtained from 16 scanners and 1236 female controls obtained from 22 scanners were included in the training samples. The top panel in Fig. 1b shows the age distribution in the training sample. A hold-out dataset comprised of controls served as a test sample to validate the accuracy of the brain age prediction model; 927 male and 1199 female controls from the same scanning sites were included. Likewise, 986 male and 1689 female MDD patients from the corresponding scanning sites were included in the MDD test sample. The two bottom panels in Fig. 1b show the age distributions across the test samples.

### Image processing and analysis

Structural T1-weighted scans of each subject were acquired at each site. To promote data sharing and to maximize the efficiency of pooling existing datasets, we used standardized protocols to facilitate harmonized image analysis and feature extraction (*N* = 153) across multiple sites (http://enigma.ini.usc.edu/protocols/imaging-protocols/). Cortical parcellations were based on the Desikan/Killiany atlas [27]. Briefly, the fully automated and validated segmentation software FreeSurfer was used to segment 14 subcortical gray matter regions (nucleus accumbens, amygdala, caudate, hippocampus, pallidum, putamen, and thalamus), 2 lateral ventricles, 68 cortical thickness, and 68 surface area measures, and total intracranial volume (ICV). Segmentations were visually inspected and statistically examined for outliers. Further details on cohort type, image acquisition parameters, software descriptions, and quality control may be found in Supplementary Table S4. Individual level structural brain measures and clinical and demographic measures from each cohort were pooled at a central site to perform the mega-analysis.

### FreeSurfer brain age prediction model

To estimate the normative brain age models, we combined the FreeSurfer measures from the left and right hemispheres by calculating the mean ((left + right)/2) of volumes for subcortical regions and lateral ventricles, and thickness and surface area for cortical regions, resulting in 77 features (Supplementary Table S5). Using a mega-analytic approach, we first estimated normative models of the association between the 77 average structural brain measures and age in the training sample of controls (separately for males and females) using ridge regression, from the Python-based *sklearn* package [28]. All brain measures were combined as predictors in a single multivariate model. To assess model performance, we performed tenfold cross-validation. To quantify model performance, we calculated the mean absolute error (MAE) between predicted brain age and chronological age. The literature suggests nonuniform age-related changes for cortical thickness, surface area, and subcortical volumes [29], which is further supported by empirical evidence showing that brain morphology is under control of distinct genetic and developmental pathways [30–33]. We therefore included all three feature modalities in our brain age prediction framework. Of note, we also tested whether reducing feature space by including only single modalities (only cortical thickness vs. cortical surface area vs. subcortical volume features) would improve model fit, but this resulted in poorer performance accuracy than combining all 77 features. Moreover, we also (1) estimated a model including left and right hemisphere features separately, (2) compared the ridge regression with other machine-learning methods, and (3) regressed features on ICV instead of including ICV as a separate feature, none of which resulted in significantly superior prediction accuracy (the results are provided in Supplementary Table S6).

### Model validation

Model performance was further validated in the test sample of controls. The parameters learned from the trained model in controls were applied to the test sample of controls and to the MDD test samples to obtain brain-based age estimates. To assess model performance in these test samples, we calculated (1) MAE, (2) Pearson correlation coefficients between predicted brain age and chronological age, and (3) the proportion of the variance explained by the model (*R*^2^). To evaluate generalizability to completely independent test samples (acquired on completely independent scanning sites), we applied the training model parameters to control subjects (males, *N* = 610; females, *N* = 720) from the ENIGMA BD working group.

### Statistical analyses

All statistical analyses were conducted in the test samples only. Brain-PAD (predicted brain-based age—chronological age) was calculated for each individual and used as the outcome variable. While different prediction models were built for males and females, the generated brain-PAD estimates were pooled for statistical analyses.

Each dependent measure of the i^th^ individual at j^th^ scanning site was modeled as follows:

1. Brain-PAD_ij_ = intercept + β_1_(Dx) + β_2_(sex) + β_3_(age) + β_4_(age^2^) + β_5_(Dx × age) + β_6_(Dx × sex) + β_7_(age × sex) + β_8_(Dx × age × sex) + U_j_ + ε_ij_
2. Brain-PAD_ij_ = intercept + β_1_(Dx) + β_2_(sex) + β_3_(age) + β_4_(age^2^) + β_5_(Dx × age) + β_6_(Dx × sex) + U_j_ + ε_ij_
3. Brain-PAD_ij_ = intercept + β_1_(Dx) + β_2_(sex) + β_3_(age) + β_4_(age^2^) + U_j_ + ε_ij_

Intercept, Dx (MDD diagnosis), sex, and all age effects were fixed. The term U_j_ and ε_ij_ are normally distributed and represent the random intercept attributed to scanning site and the residual error, respectively.

Following Le et al. [34], we posthoc corrected for the residual age effects on the brain-PAD outcome in the test samples by adding age as a covariate to our statistical models. However, we found remaining nonlinear age effects on our brain-PAD outcome [35], and included both linear and quadratic age covariates as it provided significantly better model fit to our data compared with models with a linear age covariate only (χ^2^(2) = 9.73, *p* < 0.002). For more details see Supplementary Material.

Within MDD patients, we also used linear mixed models to examine associations of brain-PAD with clinical characteristics, including recurrence status (first vs. recurrent episode), antidepressant use at time of scanning (yes/no), remission status (currently depressed vs. remitted), depression severity at study inclusion ((HDRS-17) and the Beck Depression Inventory (BDI-II)), and age of onset of depression (categorized as: early, <26 years; middle adulthood, >25 and <56 years; and late adulthood onset, >55 years). Analyses were tested two-sided and findings were false discovery rate corrected and considered statistically significant at *p* < 0.05.

Finally, to gain more insight into the importance of features for making brain age predictions we (1) calculated structure coefficients (i.e., Pearson correlations between predicted brain age and each feature) in the test samples only for illustrative purposes, (2) explored single modality (either subcortical volumes or cortical thickness or cortical surface area features) trained models, and (3) perturbed features (either subcortical volumes or cortical thickness or cortical surface area) by setting their values to zero in the test samples and examining the changes in performance [36].

## Results

### Brain age prediction performance

**Fig. 2:**
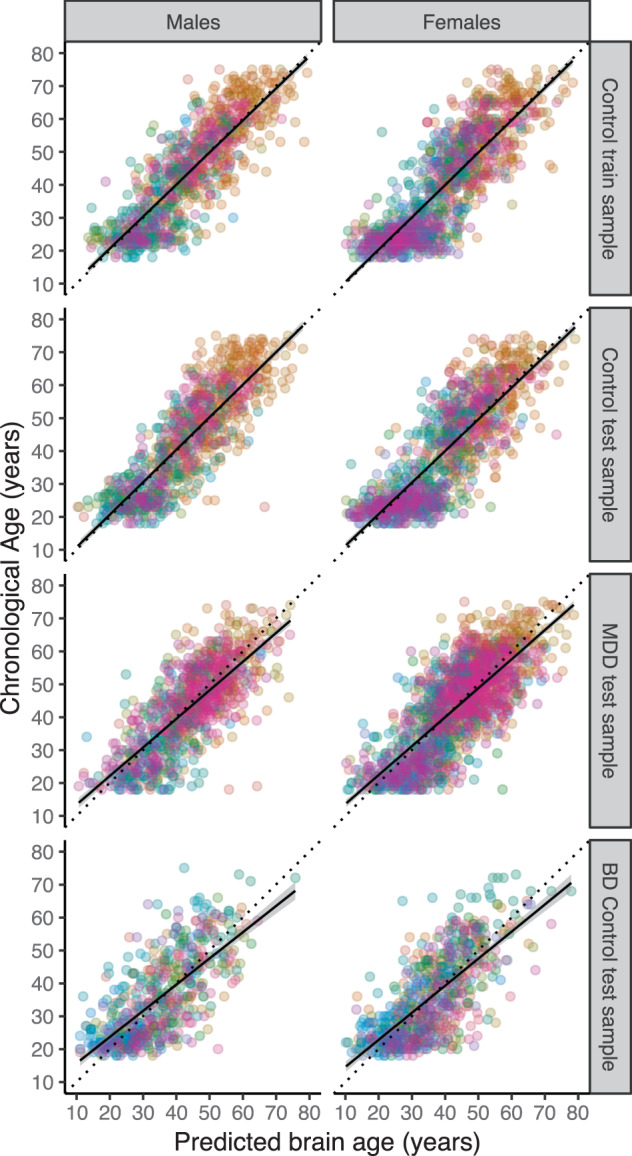
Brain age prediction based on 7 FreeSurfer subcortical volumes, lateral ventricles, 34 cortical thickness and 34 surface area measures, and total intracranial volume. The plots show the correlation between chronological age and predicted brain age in the tenfold cross-validation of the ridge regression in the control train sample, the out-of-sample validation of the test samples (controls and MDD patients) from the ENIGMA MDD working group, and generalizability to completely independent test samples (controls only) from the ENIGMA BD working group (top to bottom). The colors indicate scanning sites and each circle represents an individual subject. Diagonal dashed line reflects the line of identity (*x* = *y*).

Supplementary Fig. S1 and Supplementary Table S7 illustrate the systematic bias in brain age estimation and the correction we applied. Within the training set of controls, under cross-validation, the structural brain measures predicted chronological age with a MAE of 6.32 (SD 5.06) years in males and 6.59 (5.14) years in females. When applying the model parameters to the test samples of controls, the MAE was 6.50 (4.91) and 6.84 (5.32) years for males and females, respectively. Similarly, within the MDD group, the MAE was 6.72 (5.36) and 7.18 (5.40) years for males and females, respectively. Figure 2 shows the correlation between chronological age (*y*-axis) and predicted brain age (*x*-axis) [37] in the cross-validation training sample (males *r* = 0.85, *p* < 0.001 and females *r* = 0.854, *p* < 0.001, both *R*^2^ = 0.72), out-of-sample controls (males *r* = 0.85, *p* < 0.001; *R*^2^ = 0.72 and females *r* = 0.83, *p* < 0.001; *R*^2^ = 0.69), and MDD test samples (males *r* = 0.77, *p* < 0.001; *R*^2^ = 0.57 and females *r* = 0.78, *p* < 0.001; *R*^2^ = 0.59), and the generalization to completely independent healthy control samples of the ENIGMA BD working group (MAE = 7.49 [SD 5.89]; *r* = 0.71, *p* < 0.001; *R*^2^ = 0.45 for males and MAE = 7.26 [5.63]; *r* = 0.72, *p* < 0.001; *R*^2^ = 0.48, for females). Prediction errors were also plotted per site and age group for subjects from the ENIGMA MDD (Supplementary Figs. S2–5) and BD working group (Supplementary Figs. S6 and S7).

### Added brain aging in MDD

**Fig. 3:**
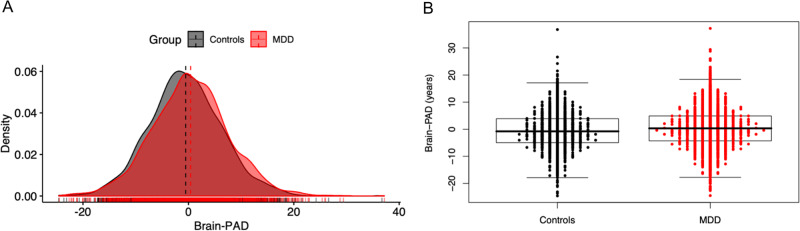
Case–control differences in brain aging. Brain-PAD (predicted brain age—chronological age) in patients with major depressive disorder (MDD) and controls. Group level analyses showed that MDD patients exhibited significantly higher brain-PAD than controls (*b* = 1.08, *p* < 0.0001), although large within-group variation and between-group overlap are observed as visualized in **a** the density plot and **b** the Engelmann–Hecker plot. The brain-PAD estimates are adjusted for chronological age, age^2^, sex, and scanning site.

Uncorrected mean brain-PAD scores were −0.20 (SD 8.44) years in the control and +0.68 years (SD 8.82) in the MDD group. Individuals with MDD showed +1.08 (SE 0.22) years higher brain-PAD than controls (*p* < 0.0001, Cohen’s *d* = 0.14, 95% CI: 0.08–0.20) adjusted for age, age^2^, sex, and scanning site (Fig. 3). In Addition, we found significant main effects for age (*b* = −0.28, *p* < 0.0001) and age^2^ (*b* = −0.001, *p* < 0.01). Our analyses revealed no significant three-way interaction between diagnosis by age and by sex, nor significant two-way interactions (diagnosis by age or diagnosis by sex). Of note, since there were no significant interactions with age or age^2^ and MDD status, and the residual age effects in the brain-PAD estimates did not influence our primary finding. Given that our model showed higher errors in individuals >60 years, we performed a sensitivity analysis by including only participants within the range of 18–60 years age. Here, we found a slightly increased effect of diagnostic group (MDD + 1.16 years [SE 0.24] higher brain-PAD than controls [*p* < 0.0001, Cohen’s *d* = 0.15, 95% CI: 0.09–0.21]).

### The relative importance of thickness features

**Fig. 4:**
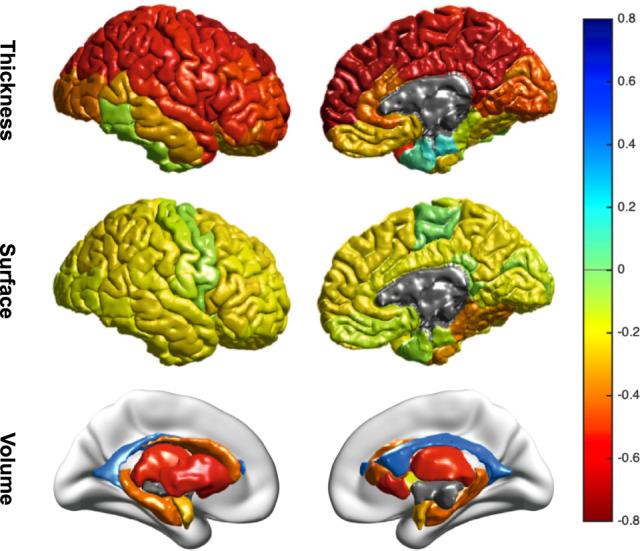
Structure coefficients of predicted brain age and FreeSurfer features across control and major depressive disorder (MDD) groups. Bivariate correlations are shown for illustrative purposes and to provide a sense of importance of features in the brain age prediction. The figure shows Pearson correlations between predicted brain age and cortical thickness features (top row), cortical surface areas (middle row), and subcortical volumes (bottom row). The negative correlation with ICV was excluded from this figure for display purposes.

All features, except the mean lateral ventricle volume, and entorhinal and temporal pole thickness showed a negative correlation with predicted brain age, and are visualized in Fig. 4. Widespread negative correlations with average cortical thickness and surface area were observed, although thickness features resulted in stronger negative correlations (mean Pearson *r* [SD]: −0.44 [0.21]) than surface area features (−0.17 [0.08]). On average, subcortical volumes were slightly less negatively correlated to predicted brain age as thickness features (−0.34 [0.34]). Single modality models and ICV performed worse than a combined model including all modalities (Supplementary Table S8). Test performance was most negatively affected by the perturbation of thickness features (Supplementary Table S9).

### Brain aging and clinical characteristics

Compared with controls, significant brain-PAD differences in years were observed in patients with a remitted disease status (+2.19 years, *p* < 0.0001, *d* = 0.18), with a current depression (+1.5 years, *p* < 0.0001, *d* = 0.18), in those that were using antidepressant medication at the time of scanning (+1.4 years, *p* < 0.0001, *d* = 0.15), medication-free depressed patients (+0.7 years, *p* = 0.0225, *d* = 0.07), patients with a late adult-onset of depression (+1.2 years, *p* = 0.01, *d* = 0.12), patients with an age of onset of MDD in mid-adulthood (+0.9 years, *p* = 0.0005, *d* = 0.11), patients with an early age of onset of depression (<26 years; +1.0 years, *p* = 0.0004, *d* = 0.11), first-episode patients (+1.2 years, *p* = 0.0002, *d* = 0.13) and recurrent depressed patients (+1.0 years, *p* = 0.0002, *d* = 0.11) (Table 1). Importantly, posthoc comparisons between the MDD subgroups did not show any significant differences (i.e., first vs. recurrent episode, antidepressant medication-free vs. antidepressant users, remitted vs. currently depressed patients, or early vs. adult vs. late age of onset of depression). Mean brain-PAD was above zero in all MDD subgroups, indicating that all MDD subgroups were estimated to be older than expected based on the brain age model compared with controls. Finally, there were no significant associations with depression severity or current depressive symptoms (self-reported BDI-II [*b* = 0.04, *p* = 0.06] or clinical-based HDRS-17 [*b* = −0.02, *p* = 0.48] questionnaires) at the time of scanning within the MDD sample.

**Table 1:** Clinical characteristics and brain aging (*N* = 2126 controls).

MDD patients vs. controls	*N*	*b (P*_FDR_ value)	SE	Cohen’s *d*	SE	95% CI
All MDD patients	2675	1.08 (<0.0001)	0.22	0.14	0.03	0.08–0.20
First-episode MDD	903	1.22 (0.0002)	0.30	0.13	0.04	0.05–0.21
Recurrent episode MDD	1648	0.97 (0.0002)	0.25	0.11	0.03	0.05–0.18
Current MDD	1786	1.47 (<0.0001)	0.28	0.18	0.04	0.11–0.26
Remitted MDD	298	2.19 (<0.0001)	0.53	0.18	0.06	0.06–0.31
AD medication-free	939	0.67 (0.0225)	0.29	0.07	0.04	−0.01 to 0.15
AD user	1717	1.36 (<0.0001)	0.26	0.15	0.03	0.09–0.22
Early-onset MDD	1035	0.98 (0.0004)	0.27	0.11	0.04	0.04–0.19
Middle adult-onset MDD	1218	0.91 (0.0005)	0.26	0.11	0.04	0.04–0.18
Late adult-onset MDD	259	1.21 (0.0107)	0.47	0.12	0.07	−0.01 to 0.25

Positive brain-PAD scores (predicted brain age—chronological age) were found for all subgroups of patients with MDD compared with controls. Regression coefficient *b* indicates the average brain-PAD difference between MDD patients and controls in years. *P* values are FDR adjusted.

*AD* antidepressant, *FDR* false discovery rate, *MDD* major depressive disorder.

## Discussion

Using a new parsimonious multisite brain age algorithm based on FreeSurfer ROIs from over 2800 males and 4100 females, we found subtle age-associated gray matter differences in adults with MDD. At the group level, patients had, on average, a +1.08 years greater discrepancy between their predicted and actual age compared with controls. Significantly larger brain-PAD scores were observed in all patient subgroups compared with controls (with Cohen’s *d* effect sizes ranging from 0.07 to 0.18), indicating that the higher brain-PAD in patients was not driven by specific clinical characteristics (recurrent status, remission status, antidepressant medication use, age of onset, or symptom severity). This study confirms previously observed advanced cellular aging in MDD at the brain level of analysis; however, it is important to mention the large within-group and small between-group variance, demonstrating that many patients did not show advanced brain aging. We were not able to investigate all potential clinical, biological, and other sources that could explain the large within-group variance of brain-PAD in MDD patients. Future studies, ideally with in-depth clinical phenotyping and longitudinal information on mental and somatic health outcomes (e.g., genomic variation, omics profiles, comorbidities, duration of illness, lifestyle, inflammation, oxidative stress, chronic diseases), are required to further evaluate the predictive value of the brain-PAD estimates, potentially by using our publicly available brain age model (https://www.photon-ai.com/enigma_brainage).

Perhaps surprisingly, we found higher brain-PAD in antidepressant users (+1.4 years depressive disorder) compared with controls and antidepressant-free patients (+0.7 years) and controls, although the difference between patient groups was not significant. Antidepressants are suggested to exert a neuroprotective effect, for example by promoting brain-derived neurotrophic factor (BDNF) [38]. However, patients taking antidepressant medication at the time of scanning likely had a more severe or chronic course of the disorder [14, 15]. Therefore, the larger brain-PAD in antidepressant users may be confounded by severity or course of the disorder. Unfortunately, the cross-sectional nature of the current study and the lack of detailed information on lifetime use, dosage and duration of use of antidepressants, do not allow us to draw any conclusions regarding the direct effects of antidepressants on brain aging. In addition, it remains to be elucidated how adaptable brain-PAD is in response to pharmacotherapy. Randomized controlled intervention studies are needed to develop an understanding of how reversible or modifiable brain aging is in response to pharmacological and nonpharmacological strategies (e.g., psychological, exercise, and/or nutritional interventions), as seen in other biological age indicators [21, 39].

Our brain-PAD difference (+1.1 years) is attenuated in contrast to earlier work showing +4.0 years of brain aging in a smaller sample of MDD patients in a study by Koutsouleris et al. (*N* = 104) [23]. However, a recent study by Kaufmann et al. found a similar effect size to ours in 211 MDD patients (18–71 years), albeit nonsignificant [25]. Although the MAE of our models (6.6 years in age range of 18–75 years) is higher than in e.g,. the study by Koutsouleris et al. (4.6 years in age range of 18–65 years), a simple calculation shows that, when scaled to covered age range, the studies show comparable MAE (0.11 vs. 0.10, respectively) [40]. As the range of possible predictions (age range) carry a strong bearing on prediction accuracy, increasingly wider ranges of outcomes become more challenging to predict [11]. Several methodological differences may underlie the inconsistencies or differences in magnitude of brain age effects in MDD, including, but not limited to (1) the use of high-dimensional features such as whole-brain gray matter maps in the Koutsouleris et al. study vs. a much lower number of input features (FreeSurfer ROIs) in our study, although the Kaufmann et al. study included multimodal parcellations and found similar brain age effects in MDD as we observed, (2) the composition of training and test data, including number of scanners in both sets, with 5 scanners included in the Koutsouleris et al. study vs. 22 in our study vs. 68 scanners in Kaufmann et al., (3) sample sizes of training and test data (*N* = 800 in training set and *N* = 104 in MDD test set in Koutsouleris et al. vs. *N* > 950 in training set and *N* > 980 in MDD test set in our current study vs. *N* > 16k training set and *N* = 211 in MDD test set in Kaufmann et al.), and (4) heterogeneity of MDD and differences in patient characteristics between the studies. The inconsistencies between brain-PAD findings in MDD might be due to any (combination) of the sources of variation outlined above and precludes a direct comparison of these studies. Unfortunately, a methodological comparison is beyond the scope of our study and beyond our capability given data access limitations within ENIGMA MDD. Nevertheless, the current results are based on the largest MDD sample to date and likely provide more precise estimates regardless of the size of the effect [41, 42].

The current findings in MDD also show lower brain aging than previously observed in SCZ (brain-PAD ranges from +2.6 to +5.5 years) [23, 40], even in the early stages of first-episode SCZ. Inconsistent findings have been reported in BD, with “younger” brain age or no differences compared with controls [11]. While the same sources of variation described above in comparing our findings with previous brain aging findings in MDD also apply here, brain abnormalities might be subtler in MDD compared with BD or SCZ. This is in line with previous ENIGMA studies in SCZ, BD, and MDD, showing the largest effect sizes of structural brain alterations in SCZ [43, 44] (highest Cohen’s *d* effect size −0.53), followed by BD [45, 46] (highest Cohen’s *d* −0.32) and MDD (highest Cohen’s *d* −0.14) [14, 15]. Conceivably more in line with MDD pathology [47], Liang et al. showed significantly higher brain-PAD in posttraumatic stress disorder (PTSD) using similar ridge regression and bias correction methods to the current paper [48]. This is consistent with similar effect sizes of structural alterations of individual brain regions observed across MDD and PTSD in large scale studies (highest Cohen’s *d* −0.17) [49].

Inflammation may be a common biological mechanism between MDD and brain aging [50]. Neuroimmune mechanisms (e.g., proinflammatory cytokines) influence biological processes (e.g. synaptic plasticity), and inflammatory biomarkers are commonly dysregulated in depression [51]. One study showed that brain-PAD was temporarily reduced by 1.1 years due to the probable acute anti-inflammatory effects of ibuprofen, albeit in healthy controls [52]. In MDD, both cerebrospinal fluid and peripheral blood interleukin (IL)-6 levels are elevated [53]. Moreover, work by Kakeda et al. demonstrated a significant inverse relationship between IL-6 levels and surface-based cortical thickness and hippocampal subfields in medication-free, first-episode MDD patients [54]. This accords with the current study that increased brain-PAD was also observed in first-episode patients compared with controls, perhaps suggesting that neuroimmune mechanisms may be chief candidates involved in the brain morphology alterations, even in the early stage of illness. Further, the age-related structural alterations in MDD may also be explained by shared underlying (epi)genetic mechanisms involved in brain development and plasticity (thereby influencing brain structure) and psychiatric illness. For instance, Aberg et al. showed that a significant portion of the genes represented in overlapping blood–brain methylome-wide association findings for MDD was important for brain development, such as induction of synaptic plasticity by BDNF [55].

In terms of individual FreeSurfer measures that contributed most to the brain age prediction, we particularly found widespread negative correlations between predicted brain age and average cortical thickness and subcortical volume, and comparably weaker correlations with surface area features (Fig. 4). We visualized these associations separately for controls and MDD patients, but findings were similar and suggest comparable structure coefficients in both groups (Supplementary Fig. S8). Notably, we did not include a spatial weight map of our brain age model, as the weights (although linear) are obtained from a multivariable model, and do not allow for a straightforward interpretation of the importance of the brain regions contributing to the aging pattern. Instead, exploratory analyses pointed out that our model relied most on the cortical thickness features in order to make good predictions. This is consistent with existing literature that supports the importance and sensitivity of cortical thickness towards aging, different from surface areas [56]. However, models including the largest feature set demonstrated the best performance (Supplementary Tables S8–10).

### Limitations and future directions

While our results are generally consistent with the existing literature on advanced or premature biological aging and major depression using other biological indicators, we also have to acknowledge some limitations. First, limited information was available on clinical characterization due to the lack of harmonization of data collection across participating cohorts. However, we provided all participating sites with their brain-PAD estimates, and encourage them to characterize brain-PAD determinants in more detail (e.g., using more in-depth phenotyping or examining associations with longitudinal outcomes). Second, we did not have access to raw individual level data and future studies could include higher-dimensional gray matter features or additional modalities such as white matter volumes, hyperintensities, and/or microstructure, or functional imaging data to examine whether model fit can be improved. However, we must also appreciate a pragmatic approach for collating data from such a large number of scanning sites. Here, we developed a parsimonious model based on FreeSurfer features collected with standardized ENIGMA extraction scripts to promote model sharing. While pooling harmonized data from many sites increases (clinical) heterogeneity, it also makes predictive models less susceptible to overfitting and more generalizable to other populations [57], even though this might have come at the cost of lower accuracy [58]. Finally, the large within-group variance regarding the brain-PAD outcome in both controls and MDD (Fig. 3), compared with the small between-group variance, renders the use of this brain aging indicator for discriminating patients and controls at the individual level difficult. As many of the MDD patients do not show advanced brain aging compared with controls, the clinical significance of the observed higher brain-PAD in MDD patients may be limited. Aberrant brain aging is not specific to MDD [11, 13, 22, 25], and it remains to be elucidated whether age-related brain atrophy is a consequence or cause of MDD. While currently brain age certainly would not constitute a viable biomarker for the diagnosis of depression based on our findings, it could potentially be used to identify those MDD patients at greater risk of poorer brain- or general health outcomes, given previous associations of older-appearing brains relating to cognitive decline, dementia, and death [59–62]. Future longitudinal studies examining the association between brain-PAD and mental, neurological, or general health outcomes specifically in individuals with MDD are required to determine whether brain-PAD could provide a clinically useful biomarker.

## Conclusions

In conclusion, compared with controls, both male and female MDD patients show advanced brain aging of around 1 year. This significant but subtle sign of advanced aging is consistent with other studies of biological aging indicators in MDD at cellular and molecular levels of analysis (i.e., telomere length and epigenetic age). The deviation of brain metrics from normative aging trajectories in MDD may contribute to increased risk for mortality and aging-related diseases commonly seen in MDD. However, the substantial within-group variance and overlap between groups signify that more (longitudinal) work including in-depth clinical characterization and more precise biological age predictor systems are needed to elucidate whether brain age indicators can be clinically useful in MDD. Nevertheless, our work contributes to the maturation of brain age models in terms of generalizability, deployability, and shareability, in pursuance of a canonical brain age algorithm. Other research groups with other available information on longitudinal mental and somatic health outcomes, other aging indicators, and incidence and/or prevalence of chronic diseases may use our model to promote the continued growth of knowledge in pursuit of useful clinical applications.

## Supplementary information

Supplementary Tables

Supplementary Materials
