# Supplementary material for: Brain aging in major depressive disorder: results from the ENIGMA major depressive disorder working group
Source: Mol Psychiatry. 2020 May 18;26(9):5124–39. doi: 10.1038/s41380-020-0754-0 (PMC8589647; doi:10.1038/s41380-020-0754-0)
Supplement: Supplementary file 1 — Supplementary Tables [file 41380_2020_754_MOESM1_ESM.docx]

**Brain aging in Major Depressive Disorder - Supplementary Tables**

**Content:**

**Supplementary Table S1.** **ENIGMA - Major Depressive Disorder Working Group Demographics.** Age (in years), and MDD patients-control breakdown per participating cohort, separately for males and females, and training and test samples.

**Supplementary Table S2. ENIGMA - Major Depressive Disorder Working Group Clinical characteristics of MDD patients.** Percentage of MDD patients using antidepressant medication, percentage of first episode and recurrent episode MDD patients, percentage of acutely depressed and remitted MDD patients, age of onset of MDD and severity of symptoms breakdown for participating cohort, separately for male and female samples.

**Supplementary Table S3. ENIGMA - Major Depressive Disorder Working Group Instrument for diagnosing Major Depressive Disorder and exclusion criteria by cohort.**

**Supplementary Table S4. ENIGMA - Major Depressive Disorder Working Group Image acquisition and processing by cohort.**

**Supplementary Table S5. A full list of the 77 gray matter FreeSurfer features included in our brain age model.** Left and right features were averaged across hemisphere.

**Supplementary Table S6. Alternative machines, kernels, and feature engineering in the brain age prediction framework.** Performance metrics in the training samples of males and females across four different machine learning algorithms/kernels are displayed here.

**Supplementary Table S7. Mean absolute error (MAE) and brain predicted age difference (brain-PAD) per age group in the overall test samples of controls and major depressive disorder (MDD) patients**.

**Supplementary Table S8. Alternative feature selection in the brain age prediction framework.** Performance metrics under 10-fold cross-validation in the training samples of males and females using features from three different modalities are displayed here. R, Pearson’s correlation; R^2^, explained variance; MAE, mean absolute error.

**Supplementary Table S9. Feature importance of modalities.** The full model includes all orginal testdata and 77 features. The rows indicate which features (subcortical volumes [8 features] or cortical thickness [34 features] or cortical surface area [34 features]) were perturbed (values set to zero) in the test samples. Test performance is most negatively affected by the perturbation of cortical thickness features. R, Pearson correlation coefficient; R2, explained variance; MAE, mean absolute error; M, males; F, females.

**Supplementary Table S10*.* A qualitative comparison between structure coefficients.** The top 10% column indicates major depressive disorder patients with the highest decile of brain-PAD values. The difference is sorted from smallest to largest and reflects the difference compared to the bottom 90% of brain-PAD values.

**Supplementary Table S1. ENIGMA - Major Depressive Disorder Working Group Demographics.** Age (in years), and MDD patients-control breakdown per participating cohort, separately for males and females, and training and test samples.

| Cohort | | Control training samples | | | | | | | |
| --- | --- | --- | --- | --- | --- | --- | --- | --- | --- |
|  |  | Males | | | | Females | | | |
|  |  | N | Age (years) | | | N | Age (years) | | |
| 1 | Barcelona | NA | NA |  | NA | 12 | 45.67 | ± | 8.85 |
| 2 | BiDirect | 109 | 51.41 | ± | 8.17 | 110 | 53.32 | ± | 8.00 |
| 3 | CLiNG | 66 | 25.86 | ± | 6.20 | 96 | 24.66 | ± | 4.94 |
| 4 | Dublin | 39 | 34.09 | ± | 9.53 | 38 | 33.83 | ± | 10.42 |
| 5 | Edinburgh (Bipolar Family Study) | NA | NA |  | NA | 22 | 22.55 | ± | 2.34 |
| 6 | FOR2107 - Marburg | 64 | 32.53 | ± | 11.02 | 100 | 33.20 | ± | 12.73 |
| 7 | FOR2107 - Münster | 20 | 27.65 | ± | 10.03 | 37 | 24.78 | ± | 6.19 |
| 8 | Houston | 18 | 39.28 | ± | 11.98 | 37 | 37.38 | ± | 13.28 |
| 9 | BRDECC London | 15 | 50.67 | ± | 8.49 | 17 | 50.88 | ± | 10.69 |
| 10 | McMaster University Mood Disorders | NA | NA |  | NA | 15 | 32.07 | ± | 11.35 |
| 11 | Melbourne | 19 | 21.16 | ± | 2.65 | 20 | 20.95 | ± | 2.48 |
| 12 | MPIP | 47 | 47.42 | ± | 12.34 | 65 | 48.07 | ± | 13.41 |
| 13 | Münster Neuroimaging Cohort | 154 | 36.28 | ± | 11.92 | 205 | 35.07 | ± | 12.08 |
| 14 | QTIM | NA | NA |  | NA | 100 | 21.68 | ± | 2.33 |
| 15 | Sao Paolo (Wellcome) | NA | NA |  | NA | 20 | 32.40 | ± | 8.15 |
| 16 | SHIP/TREND | 254 | 50.05 | ± | 13.96 | 199 | 49.65 | ± | 13.78 |
| 17 | SHIP | 115 | 54.54 | ± | 11.47 | 95 | 53.92 | ± | 11.42 |
| 18 | Stanford | 12 | 36.67 | ± | 9.41 | 19 | 36.26 | ± | 10.50 |
| 19 | Sydney | 20 | 46.65 | ± | 22.36 | 29 | 43.28 | ± | 23.27 |
|  | **Total/pooled** | 952 | 43.32 | ± | 15.24 | 1236 | 38.97 | ± | 15.68 |

*(continued on next page)*

**Supplementary Table S1.** *(continued)*

| **Cohort** | | **Control test samples** | | | | | | | | **MDD test samples** | | | | | | | |
| --- | --- | --- | --- | --- | --- | --- | --- | --- | --- | --- | --- | --- | --- | --- | --- | --- | --- |
|  |  | Males | | | | Females | | | | Males | | | | Females | | | |
|  |  | N | Age (years) | | | N | Age (years) | | | N | Age (years) | | | N | Age (years) | | |
| **1** | Barcelona | NA | NA |  | NA | 11 | 46.00 | ± | 7.90 | NA | NA |  | NA | 49 | 46.67 | ± | 7.90 |
| **2** | BiDirect | 108 | 51.33 | ± | 8.17 | 108 | 52.61 | ± | 8.06 | 231 | 47.96 | ± | 7.41 | 346 | 49.51 | ± | 7.17 |
| **3** | CLiNG | 64 | 24.84 | ± | 3.53 | 95 | 25.26 | ± | 5.53 | 23 | 38.70 | ± | 9.74 | 26 | 34.19 | ± | 12.77 |
| **4** | Dublin | 36 | 32.95 | ± | 10.86 | 32 | 34.28 | ± | 12.19 | 40 | 36.65 | ± | 9.49 | 47 | 38.08 | ± | 10.61 |
| **5** | Edinburgh (Bipolar Family Study) | NA | NA |  | NA | 20 | 22.65 | ± | 2.54 | NA | NA |  | NA | 11 | 22.82 | ± | 2.99 |
| **6** | FOR2107 - Marburg | 63 | 33.29 | ± | 12.05 | 98 | 33.47 | ± | 13.51 | 105 | 37.12 | ± | 13.58 | 171 | 37.71 | ± | 13.53 |
| **7** | FOR2107 - Münster | 17 | 26.53 | ± | 8.27 | 35 | 26.49 | ± | 11.25 | 21 | 32.43 | ± | 11.38 | 27 | 33.22 | ± | 13.47 |
| **8** | Houston | 18 | 38.22 | ± | 11.02 | 35 | 37.26 | ± | 12.51 | 23 | 39.83 | ± | 13.82 | 55 | 38.67 | ± | 13.42 |
| **9** | BRDECC London | 14 | 52.00 | ± | 5.99 | 15 | 53.47 | ± | 5.37 | 22 | 45.64 | ± | 10.01 | 47 | 48.89 | ± | 8.26 |
| **10** | McMaster University Mood Disorders | NA | NA |  | NA | 12 | 32.67 | ± | 13.27 | NA | NA |  | NA | 26 | 35.85 | ± | 13.13 |
| **11** | Melbourne | 16 | 20.75 | ± | 2.32 | 19 | 20.68 | ± | 2.21 | 27 | 20.48 | ± | 2.31 | 35 | 20.14 | ± | 1.90 |
| **12** | MPIP | 45 | 47.70 | ± | 13.22 | 63 | 49.55 | ± | 12.20 | 157 | 47.81 | ± | 13.17 | 204 | 46.80 | ± | 13.31 |
| **13** | Münster Neuroimaging Cohort | 152 | 35.23 | ± | 11.23 | 202 | 35.65 | ± | 12.68 | 115 | 36.98 | ± | 11.24 | 156 | 38.63 | ± | 12.35 |
| **14** | QTIM | NA | NA |  | NA | 97 | 21.58 | ± | 1.89 | NA | NA |  | NA | 44 | 21.69 | ± | 2.06 |
| **15** | Sao Paolo (Wellcome) | NA | NA |  | NA | 20 | 32.90 | ± | 9.53 | NA | NA |  | NA | 17 | 28.59 | ± | 7.90 |
| **16** | SHIP/TREND | 252 | 49.89 | ± | 13.95 | 199 | 49.93 | ± | 13.14 | 106 | 47.33 | ± | 9.80 | 200 | 49.34 | ± | 12.40 |
| **17** | SHIP | 111 | 54.21 | ± | 12.23 | 93 | 53.53 | ± | 11.74 | 36 | 52.06 | ± | 9.55 | 95 | 52.89 | ± | 10.96 |
| **18** | Stanford | 11 | 35.27 | ± | 11.89 | 17 | 38.35 | ± | 11.11 | 23 | 37.22 | ± | 9.59 | 33 | 36.21 | ± | 10.75 |
| **19** | Sydney | 20 | 44.80 | ± | 21.83 | 28 | 42.93 | ± | 22.29 | 57 | 38.93 | ± | 21.13 | 100 | 40.20 | ± | 20.08 |
|  | **Total/Pooled** | 927 | 43.09 | ± | 15.32 | 1199 | 39.37 | ± | 15.69 | 986 | 42.81 | ± | 13.11 | 1689 | 43.24 | ± | 14.01 |

Age reflects chronological age (mean ± SD in years). MDD, major depressive disorder. Total N=6,989.

**Supplementary Table S2.** ENIGMA - Major Depressive Disorder Working Group Clinical characteristics of MDD patients.

| **Cohort** | | Males | | Females | | Males | | Females | | Males | | Females | |
| --- | --- | --- | --- | --- | --- | --- | --- | --- | --- | --- | --- | --- | --- |
|  |  | AD  Free (%) | AD  User (%) | AD  Free (%) | AD  User (%) | First (%) | Recurrent(%) | First (%) | Recurrent(%) | Remitted  (%) | Current (%) | Remitted  (%) | Current  (%) |
| **1** | Barcelona | NA | NA | 4 | 96 | NA | NA | 31 | 69 | NA | NA | 41 | 59 |
| **2** | BiDirect | 12 | 88 | 13 | 87 | 54 | 46 | 58 | 42 | 0 | 100 | 0 | 100 |
| **3** | CLiNG | 0 | 100 | 12 | 88 | 57 | 43 | 38 | 62 | 9 | 91 | 4 | 96 |
| **4** | Dublin | 59 | 41 | 17 | 83 | 31 | 69 | 28 | 72 | 0 | 100 | 0 | 100 |
| **5** | Edinburgh (Bipolar Family Study) | NA | NA | 73 | 27 | NA | NA | NA | NA | NA | NA | NA | NA |
| **6** | FOR2107 - Marburg | 41 | 59 | 35 | 65 | 26 | 74 | 28 | 72 | 17 | 83 | 25 | 75 |
| **7** | FOR2107 - Münster | 19 | 81 | 52 | 48 | 45 | 55 | 37 | 63 | 24 | 76 | 33 | 67 |
| **8** | Houston | 100 | 0 | 100 | 0 | 29 | 71 | 23 | 77 | 0 | 100 | 5 | 95 |
| **9** | BRDECC London | 23 | 77 | 30 | 70 | 0 | 100 | 0 | 100 | NA | NA | NA | NA |
| **10** | McMaster University Mood Disorders | NA | NA | 35 | 65 | NA | NA | 35 | 65 | NA | NA | 0 | 100 |
| **11** | Melbourne | 78 | 22 | 69 | 31 | 29 | 71 | 33 | 67 | 0 | 100 | 0 | 100 |
| **12** | MPIP | 15 | 85 | 17 | 83 | 31 | 69 | 25 | 75 | 13 | 87 | 14 | 86 |
| **13** | Münster Neuroimaging Cohort | 7 | 93 | 8 | 92 | 25 | 75 | 23 | 77 | 9 | 91 | 8 | 92 |
| **14** | QTIM | NA | NA | 80 | 20 | NA | NA | NA | NA | NA | NA | NA | NA |
| **15** | Sao Paolo (Wellcome) | NA | NA | 53 | 47 | NA | NA | 33 | 67 | NA | NA | 0 | 100 |
| **16** | SHIP/TREND | 87 | 13 | 81 | 20 | 44 | 56 | 33 | 68 | NA | NA | NA | NA |
| **17** | SHIP | 78 | 22 | 83 | 17 | 58 | 42 | 55 | 45 | NA | NA | NA | NA |
| **18** | Stanford | 60 | 40 | 56 | 44 | 9 | 91 | 13 | 87 | 0 | 100 | 0 | 100 |
| **19** | Sydney | 51 | 49 | 29 | 71 | 28 | 72 | 13 | 87 | 91 | 9 | 82 | 18 |
|  | **Total/Pooled** | 33 | 67 | 37 | 63 | 37 | 63 | 34 | 66 | 13 | 87 | 15 | 85 |

Percentage of MDD patients using antidepressant (AD) medication, percentage of first episode and recurrent episode MDD patients, percentage of acutely depressed and remitted MDD patients breakdown for participating sites, separately for male and female samples. *(continued on next page)*

**Supplementary Table S2.** *(continued)*

| **Cohort** | | Males | | | Females | | | Males | | | Females | | | Males | | | Females | | |
| --- | --- | --- | --- | --- | --- | --- | --- | --- | --- | --- | --- | --- | --- | --- | --- | --- | --- | --- | --- |
|  |  | Age of Onset MDD (years) | | | | | | HDRS-17^a^ | | | | | | BDI-II^b^ | | | | | |
| **1** | Barcelona | NA |  | NA | 32.43 | ± | 11.44 | NA |  | NA | 12.61 | ± | 8.84 | NA |  | NA | NA |  | NA |
| **2** | BiDirect | 39.50 | ± | 9.97 | 37.86 | ± | 11.34 | 12.51 | ± | 6.76 | 14.33 | ± | 6.61 | NA |  | NA | NA |  | NA |
| **3** | CLiNG | 34.52 | ± | 9.99 | 26.69 | ± | 9.81 | 20.53 | ± | 4.47 | 19.33 | ± | 4.05 | 20.63 | ± | 14.47 | 22.64 | ± | 6.38 |
| **4** | Dublin | 29.29 | ± | 10.61 | 27.37 | ± | 11.29 | 23.58 | ± | 4.54 | 23.55 | ± | 5.37 | 21.42 | ± | 5.48 | 19.75 | ± | 4.43 |
| **5** | Edinburgh (Bipolar Family Study) | NA |  | NA | 22.50 | ± | 2.78 | NA |  | NA | 7.20 | ± | 6.86 | NA |  | NA | NA |  | NA |
| **6** | FOR2107 - Marburg | 26.68 | ± | 13.46 | 26.48 | ± | 12.58 | 8.43 | ± | 6.18 | 8.13 | ± | 6.52 | 19.76 | ± | 10.28 | 18.42 | ± | 11.54 |
| **7** | FOR2107 - Münster | 25.86 | ± | 10.12 | 24.93 | ± | 11.31 | 8.86 | ± | 7.23 | 9.15 | ± | 7.61 | 18.25 | ± | 12.28 | 15.26 | ± | 11.70 |
| **8** | Houston | 22.65 | ± | 9.80 | 21.12 | ± | 11.13 | 11.96 | ± | 8.50 | 9.67 | ± | 7.71 | 14.79 | ± | 13.85 | 17.49 | ± | 15.61 |
| **9** | BRDECC London | 17.56 | ± | 5.73 | 21.89 | ± | 10.42 | NA |  | NA | NA |  | NA | 17.11 | ± | 13.43 | 17.51 | ± | 11.46 |
| **10** | McMaster University Mood Disorders | NA |  | NA | 24.54 | ± | 9.76 | NA |  | NA | 12.00 | ± | 6.54 | NA |  | NA | NA |  | NA |
| **11** | Melbourne | 17.68 | ± | 2.63 | 17.03 | ± | 3.10 | NA |  | NA | NA |  | NA | NA |  | NA | NA |  | NA |
| **12** | MPIP | 35.28 | ± | 13.75 | 32.99 | ± | 12.89 | 25.99 | ± | 6.29 | 26.91 | ± | 7.10 | 13.44 | ± | 9.63 | 14.45 | ± | 11.31 |
| **13** | Münster Neuroimaging Cohort | 29.41 | ± | 11.81 | 30.14 | ± | 11.79 | 21.18 | ± | 6.79 | 21.81 | ± | 7.81 | 23.35 | ± | 9.31 | 26.29 | ± | 11.10 |
| **14** | QTIM | NA |  | NA | 18.57 | ± | 2.86 | NA |  | NA | NA |  | NA | NA |  | NA | NA |  | NA |
| **15** | Sao Paolo (Wellcome) | NA |  | NA | NA |  | NA | NA |  | NA | 15.64 | ± | 9.54 | NA |  | NA | NA |  | NA |
| **16** | SHIP/TREND | 35.84 | ± | 12.85 | 35.96 | ± | 14.44 | NA |  | NA | NA |  | NA | 12.05 | ± | 8.24 | 12.53 | ± | 8.02 |
| **17** | SHIP | 39.03 | ± | 12.90 | 37.67 | ± | 12.76 | NA |  | NA | NA |  | NA | 11.81 | ± | 9.37 | 12.01 | ± | 10.81 |
| **18** | Stanford | 20.43 | ± | 9.34 | 18.84 | ± | 9.25 | NA |  | NA | NA |  | NA | 29.13 | ± | 10.90 | 22.78 | ± | 8.29 |
| **19** | Sydney | 27.76 | ± | 19.43 | 24.76 | ± | 14.33 | 11.55 | ± | 6.47 | 12.53 | ± | 7.09 | NA |  | NA | NA |  | NA |
|  | **Total/Pooled** | 32.49 | ± | 13.71 | 30.97 | ± | 13.65 | 15.28 | ± | 8.95 | 14.98 | ± | 9.05 | 17.74 | ± | 10.87 | 17.32 | ± | 11.75 |

Age of onset of MDD and severity of symptoms breakdown for participating sites, separately for male and female samples.

^a^ Measured with the Hamilton Depression Rating Scale (HDRS-17; range: 0-52)

^b^ Measured with the Beck Depression Inventory (BDI-II; range: 0-63)

**Supplementary Table S3.** ENIGMA - Major Depressive Disorder Working Group Instrument for diagnosing Major Depressive Disorder and exclusion criteria by site.

| Cohort | Country | Diagnosis measurment | Sample characteristics/Inclusion criteria | Exclusion criteria |
| --- | --- | --- | --- | --- |
| Barcelona | **Spain** | DSM-IV-TR acc. to CIDI-interview and HAMD | Outpatients with MDD diagnosis (DSM-IV-TR), with a first episode, recurrent MDD or chronic MDD (TRD) age 18-65 | The exclusion criteria for healthy participants were. lifetime psychiatric diagnoses, first-degree relatives with psychiatric diagnoses and clinically significant physical or neurological illnesses. Axis I comorbidity according to DSM-IV-TR criteria was an exclusion criteria for all participants. |
| BiDirect | **Germany** | M.I.N.I. Neuropsychiatric Interview, IDS, HAMD, CESD, ICD-10 | Patients hospitalized for a first or recurrent episode of depression, population controls randomly selected in city registry | dementia, addiction |
| Edinburgh (Bipolar Family Study) | **Scotland** | SCID interview | The MDD group were originally people with a FHx of bipolar disorder | MDD subjects: presence of other axis I diagnoses. Control subjects: no medical history, including neurological and psychiatric history, as well as no previous or actual use of psychotropic medication All subjects: any major neurological disorder, learning disability, or any history of head injury that included loss of consciousness and any contraindications to MRI. |
| BRCDECC London | **England** | SCAN interview | Community based or outpatients, none were inpatients. MDD subjects: Less than two depressive episodes of at least moderate severity. Did not meet DSM-IV diagnostic criteria for recurrent major depressive disorder. Control group participants were clinically interviewed to ensure they had never experienced depressive symptoms.  Exclusion criteria for all participants were for contraindications to MRI; other exclusion criteria were a diagnosis of neurological disorder, head injury leading to loss of consciousness or conditions known to affect brain structure or function (including alcohol or substance misuse), ascertained during clinical interview. Potential participants were also excluded if they or a first-degree relative had ever fulfilled criteria for mania, hypomania, schizophrenia or mood-incongruent psychosis. | Contraindications to MRI, diagnosis of neurological disorder, head injury leading to loss of consciousness or conditions known to affect brain structure or function (including alcohol or substance misuse), if they or a first-degree relative had ever fulfilled criteria for mania, hypomania, schizophrenia or mood-incongruent psychosis. |
| CliNG | **Germany** | ICD-10 interview | Patients met the diagnostic criteria for major depressive disorder according to ICD-10 classification standards and were aged between 18 and 60 years. | Exclusion criteria for MDD subjects were neurological and severe other medical conditions (in particular those that could be related to affective symptoms), lifetime diagnosis of substance dependence, substance abuse during the last month, cannabis abuse during the last 2 weeks, mental retardation as well as past or actual presence of other axis I diagnoses with exception of anxiety disorders. Exclusion criteria for control subjects were neurological, psychiatric and severe other medical conditions, lifetime diagnosis of substance dependence, substance abuse during the last month, cannabis abuse during the last 2 weeks, previous or actual use of psychotropic medication, and mental retardation. |
| Dublin | **Ireland** | SCID-1 interview |  | MDD subjects: comorbid psychiatric disorders (Axis I or Axis II, other than MDD), Treatment with antipsychotics or mood stabilizers, age 65, Control subjects: no Axis-I diagnosis, no medication use. All subjects: history of neurological or other severe medical illness, head injury or severe substance abuse in their lifetime history and general MRI contraindications. |
| FOR2107 - Marburg | **Germany** | SCID-1 | Participants recruited by means of public advertisement and from the inpatient services. Inclusion criteria: age 18-65 years; patients were diagnosed with major depressive disorder by SCID-Interview, currently depressed or remitted. | Exclusion criteria all: any MRI contraindications; any neurological abnormalities. Exclusion criteria controls: any current or former psychiatric disorder; Exclusion criteria patients: substance dependence or current benzodiazepine treatment (wash out of at least three half-lives before study participation)" |
| FOR2017 - Münster | **Germany** | SCID-1 | Participants recruited by means of public advertisement and from the inpatient services. Inclusion criteria: age 18-65 years; patients were diagnosed with major depressive disorder by SCID-Interview, currently depressed or remitted. | Exclusion criteria all: any MRI contraindications; any neurological abnormalities. Exclusion criteria controls: any current or former psychiatric disorder; Exclusion criteria patients: substance dependence or current benzodiazepine treatment (wash out of at least three half-lives before study participation)" |
| Houston | **USA** | SCID interview | Outpatients | MDD subjects: age below 18; lifetime or current diagnosis of psychotic disorder, or bipolar I or II disorder; substance abuse/dependence in 6 months prior to study inclusion; current major medical problems. Control subjects: age below 18; current major medical problems; current psychiatric or neurologic disorder; history of psychiatric disorders in a first-degree relative; current major medical problems. Both groups: MRI contra-indications |
| McMaster University Mood Disorders | **Canada** | SCID | Outpatients | Comorbid Axis 1 disorders excluded, including for example, psychosis, bipolar, PTSD substance dependence or current active eating disorder. Exclusion criteria included: i) treatment with anti-cholinergic or typical (first generation) anti-psychotic medication; ii) electroconvulsive therapy (ECT) or transcranial magnetic stimulation (TMS) within the past year; iii) a history of substance dependence or significant and recent (< 1 year) substance abuse; iv) a history (within the past 12 months) of an endocrine or other medical disorder known to adversely affect cognition (e.g., Cushing’s, uncontrolled diabetes, seizure disorder); and v) English comprehension lower than a grade 6 reading level. |
| Melbourne | **Australia** | SCID interview | Youth depression sample: 15-25 years of age. Recruited as part of 2 large RCTs (incl. YoDA-C - Davey et al., 2014; Trials) and scanned prior to treatment randomisation. 60 patients unmedicated (YoDA-C). | MDD subjects: lifetime or current SCID-I diagnosis of psychotic disorder, or bipolar I or II disorder. Control subjects: any SCID-I diagnosis or medication use. Both groups: Acute or unstable medical disorder; general MRI contraindications |
| MPIP | **Germany** | M-CIDI/SCAN interview | M. A. R. S. sample: both first and recurrent episodes; RUD sample: only recurrent episodes with some patients scanned in remission | Munich Antidepressant Response Signature (MARS) study MDD subjects (clinical consensus diagnosis or M-CIDI (since 2008)): depressive syndromes secondary to any medical or neurological condition (e. g., intoxication, drug abuse, stroke), the presence of manic, hypomanic or mixed affective symptoms, lifetime diagnosis of alcohol dependence, illicit drug abuse or the presence of severe medical conditions (e.g., ischemic heart disease). Patients with bipolar depression were excluded for the current MR study. Control subjects: age > 65, MMSE<27, presence of severe somatic diseases or lifetime history of the following axis I disorders as assessed by the M-CIDI interview: alcohol dependence, drug abuse or dependence, possible psychotic disorder, mood disorder, anxiety disorder including OCD and PTSD, somatoform disorder, dissociative disorder NOS, and eating disorder 2. Recurrent unipolar depression (RUD) study: MDD subjects (SCAN interview): presence of manic episodes, mood incongruent psychotic symptoms, the presence of a lifetime diagnosis of intravenous drug abuse and depressive symptoms only secondary to alcohol or substance abuse or to medical illness or medication.Control subjects: presence of severe somatic diseases or life-time history of anxiety and affective disorders according to the Composite International Diagnostic-Screener (CIDI-S). All subjects: gross incidental MR findings such as territorial infarction, tumor, hydrocephalus, malformations and anatomical deviations (e.g. enlarged ventricles) that prevent appropriate image processing were additional exclusion criteria. 3. MR images of 9 additional controls acquired at the LMU, Munich, meeting equivalent criteria as the RUD control sample were included. |
| Münster Neuroimaging Cohort | **Germany** | SCID interview | Participants recruited by means of public advertisement and from the inpatient services. Inclusion criteria: age 16-65 years; patients were diagnosed with major depressive disorder by SCID-Interview | MDD subjects: presence of bipolar disorder, schizoaffective disorders and schizophrenia; substancerelated disorders or current benzodiazepine treatment (wash out of at least three half-lives before study participation), and former electroconvulsive therapy. Control subjects: any current or former psychiatric disorder. Both groups: any neurological abnormalities, MRI contra-indications |
| QTIM | **Australia** | CIDI interview | Retrospective questionnaire about depression episodes combined with an MRI study. The best described MDD episode is defined as the worst one (according to individuals). We have up to 5 supplementary episodes (briefly) described. Sample composed of twins and relatives. Population-based sample | MDD subjects: presence of axis-I disorders other than MDD and anxiety disorders Control subjects: antidepressant use, psychiatric disorders All subjects: relatedness between subjects, left handedness, history of neurological or other severe medical illness, head injury or current or past diagnosis of substance abuse, use of cognition affecting medication and general MRI contraindications |
| Sao Paulo (Wellcome) | **Brasil** | Hamilton Rating Scale for Depression (HRSD) | Population-based study of incident (first-episode) psychosis in outpatient services. All subjects we provided were diagnosed with psychotic depression (and not schizophrenia, bipolar disorder or other psychotic diagnoses). | People with psychotic disorders due to a general medical condition or substance-induced psychosis were excluded. Additional exclusion criteria were: (a) history of head injury; (b) presence of neurological disorders or any organic disorders that could affect the central nervous system; and (c) contraindications for MRI. Exclusion cri- teria specific for the control group were personal history of psychosis or other Axis I disorders, except substance misuse or mild anxiety disorders. |
| SHIP | **Germany** | M-CIDI interview | Population based longitudinal cohort study | MDD subjects: presence of axis-I disorders other than MDD, anxiety disorders, conversion, somatization and eating disorder. Control subjects: no lifetime diagnosis of depression, no antidepressiva, and severity index=0 All subjects: We removed subjects with medical conditions (e.g. a history of cerebral tumor, stroke, Parkinson’s diseases, multiple sclerosis, epilepsy, hydrocephalus, enlarged ventricles, pathological lesions) or due to technical reasons (e.g. severe movement artifacts or inhomogeneity of the magnetic field). |
| SHIP/TREND | **Germany** | M-CIDI interview | Population based longitudinal cohort study | MDD subjects: no special exclusion criteria Control subjects: no lifetime diagnosis of depression, no antidepressiva, and severity index=0 All subjects: We removed subjects with due to medical conditions (e.g. a history of cerebral tumor, stroke, Parkinson’s diseases, multiple sclerosis, epilepsy, hydrocephalus, enlarged ventricles, pathological lesions) or due to technical reasons (e.g. severe movement artifacts or inhomogeneity of the magnetic field). |
| Stanford | **USA** | SCID interview | Community-based DSM-diagnosed sample | MDD subjects: presence of axis-I disorders other than MDD, anxiety and eating disorders . Control subjects: control individuals did not meet diagnostic criteria for any current psychiatric. Both groups: alcohol / substance abuse or dependence within six months prior to MRI scanning, history of head trauma with loss of consciousness > 5 min, aneurysm, or any neurological or metabolic disorders that require ongoing medication or that may affect the central nervous system (including thyroid disease, diabetes, epilepsy or other seizures, or multiple sclerosis), MRI contraindications, or bad MRI data (e.g., extreme movement). |
| Sydney | **Australia** | SCID interview |  | MDD subjects: presence of axis-I disorders other than MDD, panic disorder, social anxiety disorder, or generalized anxiety disorder. Control subjects: no Axis-I diagnosis, no medication use. Exclusion criteria for all subjects included medical instability (as determined by a psychiatrist), history of neurological disease (e.g. tumour, head trauma, epilepsy), medical illness known to impact cognitive and brain function (e.g. cancer), intellectual and/or developmental disability and insufficient English for neuropsychological assessment. All subjects were asked to abstain from drug or alcohol use for 48 hours prior to testing and informed about a drug screen protocol. |

**Supplementary Table S4.** ENIGMA - Major Depressive Disorder Working Group Image acquisition and processing by cohort.

| Cohort | Country | Scanner type | Sequence T1 | FreeSurfer version | Slice orientation | Operating system |
| --- | --- | --- | --- | --- | --- | --- |
| Barcelona | **Spain** | 3T Philips Achieva | 3D MPRAGE images (Whole-brain T1-weighted); TR=6.7ms, TE=3.2ms; 170 slices, voxel size 0.89X0.89X1.2 mm. Image dimensions 288X288X170; field of view: 256X256X204; slice thickness: 1.2 mm; with a sagittal slice orientation, T1 contrast enhancement, flip angle: 8º, grey matter as a reference tissue, ACQ matrix MXP = 256X240 and turbo-field echo shots (TFE) = 218. | 6 | Sagittal | Scientific Linux 5 |
| BiDirect | **Germany** | 3 T Philips Intera scanner | 3D T1-weighted turbo field echo images were collected with a the following parameters: TR = 7.26, TE = 3.56, 9° flip angle, 160 sagittal slices, matrix dimension 256 x 256, FOV = 256 x 256mm, 2mm slice thickness (reconstructed to 1mm) and a resulting voxel size of 1x1x1mm | 5.3 | Sagittal |  |
| Edinburgh (Bipolar Family Study) | **Scotland** | 1.5T GE Signa | T1-weighted sequence. TR=500 msec; TE=4 msec; flip angle 8°; matrix 192 x 192; 180 slices; voxel size 1.25 mm x 1.25 mm x 1.20 mm; FOV=24, phase FOV 1 | 5.3 | Coronal | linux 6, x86_64, kernel 2.6.32 |
| BRCDECC London | **England** | 1.5T GE Signa HDx | ADNI-1 MPRAGE pulse sequence (details at http://adni.loni.ucla.edu/research/protocols/mri-protocols/) | 5.3 | Sagittal | Linux-centos4_x86_64 |
| CliNG | **Germany** | 3T Siemens Tim Trio | T1-weighted 3D MPRAGE; TR/TE/TI/FA=2250 ms/3.26 ms/900 ms/9°; image matrix = 256 x 256; 192 sagittal slices; voxel size= 1 mm3 | 5.3 | Sagittal | Linux |
| Dublin | **Ireland** | 3T Phillips Achieva; 1.5T Siemens Vision | 3T: A sagittal T1 3D TFE was used to scan all participants. TR=8.5 msec; TE=3.9 msec; FOV = 256 mm, AP: 256 mm, RL: 160 mm; matrix: 256×256. 1.5T: 3D-MPRAGE T1-weighted sequence. TR=11.6 msec; TE=4.9 msec; FOV=230 mm; matrix 512 x 512, slice thickness: 1.5 mm. | 5.3 | Sagittal (3T), Coronal (1.5T) | Mac OS |
| FOR2107 - Marburg | **Germany** | 3T Siemens Magnetom TiroTim syngo MR B17 | Sequence: 3D T1-weighted magnetization prepared rapid acquisition gradient echo (MPRAGE) - Sagittal Acquisition Direction, # of Slices 176, 0.5mm Slice Gap, 1.0x1.0x1.0 Voxel Size (mm3), TI 900 ms, TE 2.26 ms, TR 1900 ms, Flip Angle 9. | 5.3 | Sagittal | Red Hat Enterprise Linux Server release 5.11 (Tikanga) |
| FOR2017 - Münster | **Germany** | 3T Siemens PRISMA | Sequence: 3D T1-weighted magnetization prepared rapid acquisition gradient echo (MPRAGE). - Sagittal Acquisition Direction, # of Slices 192, 0mm Slice Gap, 1.0x1.0x1.0 Voxel Size (mm3), TI 900 ms, TE 2.28 ms, TR 1900 ms, Flip Angle 8 | 5.3 | Sagittal | Red Hat Enterprise Linux Server release 5.11 (Tikanga) |
| Houston | **USA** | subjects in 20000s: 1.5 T Philips Medical Systems Gyroscan Intera; subjects in 30000s: 3T Siemens Allegra | Subjects in the 20000s: Fast field echo sequence- repetition time (TR) = 24 ms, echo time (TE) = 4.99 ms, flip angle = 40°, slice thickness = 1 mm, matrix size = 256 × 256 and 150 slices. Subjects in 30000s: MPRAGE- repetition time (TR) = 1750 ms, echo time (TE) = 4.39 ms, flip angle = 8°, slice thickness = 1 mm, matrix size = 208 × 256 and 160 slices. | 5.3 | Subjects in 20000s: Sagittal; Subjects in 30000s: Transverse | Fedora 19 |
| McMaster University Mood Disorders | **Canada** | 1.5T (GE); 3T(GE) | 1.5-T. Sigma GE Genesis-based Echo-Speed scanner running version 5.7 software and using a standard 30-cm circularly polarized head coil. Sagittal anatomic images were acquired by using a 3D/FSPGR/20 sequence (flip angle=20; echo delay time in-phase (TE), minimum repetition time (TR)=300 ms; inversion recovery=300 ms; matrix=512x256; field of view (FOV)=24 cm; scan thickness=1.2 mm). 3-T MRI Sigma GE Genesis (General Electric Medical Systems, Milwaukee, WI). Sagittal T-1 weighted images were acquired using a 3D FSPGR-IR sequence, (TR/TE=10.3/2.1 ms; flip angle=20; inversion time=300; matrix=512x256; FOV=24; and slice thickness=1.2 mm. | 5 |  |  |
| Melbourne | **Australia** | 3T GE Signa Excite | 3D BRAVO sequence 140; TR=7900 ms; TE=3000 ms; flip angle=13º; FOV=256 mm; matrix=256 x 256 | 5.3 | Axial | Linux Debian x86 64 |
| MPIP | **Germany** | 1.5T GE and Siemens (the latter: only few cases) | #1: T1-weighted SPGR sagittal 3D volume. TR=1030 msec; TE=3.4 msec; 124 slices; matrix=256x256; FOV=23.0x23.0 cm2; voxel size=0.8975 mm x0.8975 mm x 1.2- 1.4 mm; flip angle=90°; birdcage resonator. #2: same scanner as #1, platform update Signa Excite, sagittal T1-weighted (spin echo sequence, TR=9.7 msec, TE=2.1 msec; FOV=25.0x25.0 cm2, voxel size=0.875 mm x0.875 mm x1.2 mm, 124- 132 slices, flip angle=90°. #3: Siemens 1.5 Tesla, Vario, 3D MPRAGE, TR=11.6 msec; TE=4.9 msec; FOV 23x23 cm2; matrix 512x512; 126 axial slices; voxel site 0.45 mm x 0.45 mm x 1.5 mm. (only N=2 subjects) | 5.3 | 1.5 GE: sagittal. 1.5 Siemens: axial | Linux 2.6.37.1-1.2- desktop x86_64 |
| Münster Neuroimaging Cohort | **Germany** | 3T Philips Gyroscan Intera | 3D fast gradient echo sequence (turbo field echo), repetition time = 7.4 milliseconds, echo time = 3.4 milliseconds, flip angle = 9°, two signal averages, inversion prepulse every 814.5 milliseconds, acquired over a field of view of 256 (feet -head [FH]) × 204 (anterior -posterior [AP]) × 160 (right -left [RL]) mm, phase encoding in AP and RL direction, reconstructed to cubic voxels of .5 mm × .5 mm × .5 mm | 5.3 | Sagittal | Red Hat Enterprise Linux Server release 5.11 (Tikanga) |
| QTIM | **Australia** | Bruker 4T Wholebody MRI | 3D T1 weighted sequence. TR=1500 msec; TE=3.35 msec; flip angle=8°, 256 or 240 (coronal or sagittal) slices, FOV=240 mm, matrix 256x256x256 (or 256x256x240) | 5.1 | Coronal, then sagittal following software upgrade. | Linux- centos4_x86_64- stable-pub-v5.1.0 |
| Sao Paulo (Wellcome) | **Brasil** | 1.5T General Eletric (GE) | Imaging data were acquired using two MRI scanners (at the Clinics Hospital of the University of Sa ̃ o Paulo 1.5 T GE Signa scanner, General Electric, Milwaukee Wisconsin, USA). T1-SPGR sequence providing 124 contiguous slices, voxel size 0.8660.8661.5 mm, echo time 5.2 ms, resolution time 21.7 ms, flip angle 20, field of vision 22, matrix 256x192) | 5.3 |  |  |
| SHIP | **Germany** | 1.5T Siemens Avanto | 3D T1-weighted (MP-RAGE/ axial plane); TR=1900 msec; TE=3.4 msec; Flip angle=15°; voxel size 1 mm x 1 mm x 1 mm | 5.3 (cortical), 5.1 (subcortical) | Axial | Centos6_x86_64 |
| SHIP/TREND | **Germany** | 1.5T Siemens Avanto | 3D T1-weighted (MP-RAGE/ axial plane); TR=1900 msec; TE=3.4 msec; Flip angle=15°; voxel size 1 mm x 1 mm x 1 mm | 5.3 (cortical), 5.1 (subcortical) | Axial | Centos6_x86_64 |
| Stanford | **USA** | 1.5T GE Signa Excite | Whole-brain T1-weighted images were collected using a spoiled gradient echo (SPGR) pulse sequence (116 sagittal slices; through-plane resolution = 1.5 mm; in-plane resolution = 0.86 x 0.86 mm; flip angle = 15 degrees; repetition time [TR] = 8.3-10.1 ms; echo time [TE] = 1.7-3.0; inversion time [TI] = 300 ms; matrix = 256 x 192). | 5.3 | Sagittal | Linux-centos6_x86_64 |
| Sydney | **Australia** | 3T GE MR750 | 3D T1-weighted sequence. TR=7.2 msec; TE=2.78 msec; matrix =256; FOV=240; No. slices=196; thick=0.9mm; inplane resolution=0.9375 | 5.1 but rerunning it for 5.3 | Coronal | Linux_Ubuntu16.04 lts 64bit |

**Supplementary Table S5.** A full list of the 77 gray matter FreeSurfer features included in our brain age model.

| **Modality** | **Feature** | **Anatomical ROI** |  | 39 | M_supramarginal_thickavg |
| --- | --- | --- | --- | --- | --- |
|  | 1 | ICV |  | 40 | M_frontalpole_thickavg |
| **Subcortical volume** | 2 | Mvent |  | 41 | M_temporalpole_thickavg |
|  | 3 | Mthal |  | 42 | M_transversetemporal_thickavg |
|  | 4 | Mcaud |  | 43 | M_insula_thickavg |
|  | 5 | Mput | **Mean cortical surface area** | 44 | M_bankssts_surfavg |
|  | 6 | Mpal |  | 45 | M_caudalanteriorcingulate_surfavg |
|  | 7 | Mhippo |  | 46 | M_caudalmiddlefrontal_surfavg |
|  | 8 | Mamyg |  | 47 | M_cuneus_surfavg |
|  | 9 | Maccumb |  | 48 | M_entorhinal_surfavg |
| **Mean cortical thickness** | 10 | M_bankssts_thickavg |  | 49 | M_fusiform_surfavg |
|  | 11 | M_caudalanteriorcingulate_thickavg |  | 50 | M_inferiorparietal_surfavg |
|  | 12 | M_caudalmiddlefrontal_thickavg |  | 51 | M_inferiortemporal_surfavg |
|  | 13 | M_cuneus_thickavg |  | 52 | M_isthmuscingulate_surfavg |
|  | 14 | M_entorhinal_thickavg |  | 53 | M_lateraloccipital_surfavg |
|  | 15 | M_fusiform_thickavg |  | 54 | M_lateralorbitofrontal_surfavg |
|  | 16 | M_inferiorparietal_thickavg |  | 55 | M_lingual_surfavg |
|  | 17 | M_inferiortemporal_thickavg |  | 56 | M_medialorbitofrontal_surfavg |
|  | 18 | M_isthmuscingulate_thickavg |  | 57 | M_middletemporal_surfavg |
|  | 19 | M_lateraloccipital_thickavg |  | 58 | M_parahippocampal_surfavg |
|  | 20 | M_lateralorbitofrontal_thickavg |  | 59 | M_paracentral_surfavg |
|  | 21 | M_lingual_thickavg |  | 60 | M_parsopercularis_surfavg |
|  | 22 | M_medialorbitofrontal_thickavg |  | 61 | M_parsorbitalis_surfavg |
|  | 23 | M_middletemporal_thickavg |  | 62 | M_parstriangularis_surfavg |
|  | 24 | M_parahippocampal_thickavg |  | 63 | M_pericalcarine_surfavg |
|  | 25 | M_paracentral_thickavg |  | 64 | M_postcentral_surfavg |
|  | 26 | M_parsopercularis_thickavg |  | 65 | M_posteriorcingulate_surfavg |
|  | 27 | M_parsorbitalis_thickavg |  | 66 | M_precentral_surfavg |
|  | 28 | M_parstriangularis_thickavg |  | 67 | M_precuneus_surfavg |
|  | 29 | M_pericalcarine_thickavg |  | 68 | M_rostralanteriorcingulate_surfavg |
|  | 30 | M_postcentral_thickavg |  | 69 | M_rostralmiddlefrontal_surfavg |
|  | 31 | M_posteriorcingulate_thickavg |  | 70 | M_superiorfrontal_surfavg |
|  | 32 | M_precentral_thickavg |  | 71 | M_superiorparietal_surfavg |
|  | 33 | M_precuneus_thickavg |  | 72 | M_superiortemporal_surfavg |
|  | 34 | M_rostralanteriorcingulate_thickavg |  | 73 | M_supramarginal_surfavg |
|  | 35 | M_rostralmiddlefrontal_thickavg |  | 74 | M_frontalpole_surfavg |
|  | 36 | M_superiorfrontal_thickavg |  | 75 | M_temporalpole_surfavg |
|  | 37 | M_superiorparietal_thickavg |  | 76 | M_transversetemporal_surfavg |
|  | 38 | M_superiortemporal_thickavg |  | 77 | M_insula_surfavg |

Left and right features were averaged across hemisphere.

**Supplementary Table S6.** Alternative machines, kernels, and feature engineering in the brain age prediction framework.

|  | **Alternative method** | **R** | | **R^2^** | | **MAE** | |
| --- | --- | --- | --- | --- | --- | --- | --- |
|  |  | Male training sample | Female training sample | Male training sample | Female training sample | Male training sample | Female training sample |
| **1** | **Ridge regression** | 0.85 | 0.85 | 0.72 | 0.72 | 6.32 | 6.59 |
| **2** | **SVR linear** | 0.85 | 0.84 | 0.72 | 0.71 | 6.86 | 6.91 |
| **3** | **SVR RBF** | 0.85 | 0.87 | 0.73 | 0.75 | 6.50 | 6.09 |
| **4** | **RFR** | 0.79 | 0.80 | 0.67 | 0.64 | 6.81 | 7.22 |
| **5** | **ROIs regressed on ICV** | 0.83 | 0.84 | 0.68 | 0.70 | 6.67 | 6.81 |
| **6** | **Left and right hemi** | 0.85 | 0.84 | 0.72 | 0.70 | 6.37 | 6.75 |

Performance metrics in the training samples of males and females across different machine learning algorithms/kernels and feature engineering are displayed here. Models 1-4 show alternative machines and kernels that included the same 77 features. Model 5 features included 76 individual ROIs regressed on ICV, instead of having ICV included as a separate feature. Model 6 included separate features for left and right hemispheres, instead of an average across hemispheres. R, Pearson’s correlation; R2, explained variance; MAE, mean absolute error; SVR, support vector regression; RBF, radial basis function; RFR, Random Forest Regression; ROI, regions of interest; ICV, intracranial volume; hemi, hemisphere.

**Supplementary Table S7.** Mean absolute error (MAE) and brain predicted age difference (brain-PAD) per age group.

| **Age group** | **Brain-PAD** | | **MAE** | |
| --- | --- | --- | --- | --- |
|  | **Male test samples**  **(N=2,256)** | **Female test samples**  **(N=3,370)** | **Male test samples**  **(N=2,256)** | **Female test samples**  **(N=3,370)** |
| **18-27 years** | 5.56 (7.91) | 4.65 (7.72) | 7.40 (6.22) | 7.11 (5.53) |
| **28-37 years** | 3.10 (6.97) | 3.65 (7.62) | 6.11 (4.56) | 6.77 (5.04) |
| **38-47 years** | 1.34 (7.41) | 1.75 (7.95) | 5.90 (4.66) | 6.49 (4.90) |
| **48-57 years** | -2.37 (7.13) | -3.18 (7.61) | 6.15 (4.30) | 6.61 (4.93) |
| **58-67 years** | -5.06 (7.30) | -6.14 (7.76) | 7.07 (5.38) | 7.93 (5.92) |
| **68-75 years** | -9.51 (6.77) | -9.55 (8.29) | 10.00 (6.02) | 10.47 (7.08) |

Values were calculated in the overall test samples of controls and major depressive disorder (MDD) patients.

**Supplementary Table S8.** Alternative feature selection in the brain age prediction framework.

| **Ridge Regression** | **R** | | **R^2^** | | **MAE** | |
| --- | --- | --- | --- | --- | --- | --- |
|  | Male training sample | Female training sample | Male training sample | Female training sample | Male training sample | Female training sample |
| **Subcortical volumes and ICV** | 0.75 | 0.72 | 0.56 | 0.51 | 8.01 | 8.95 |
| **Cortical thickness and ICV** | 0.78 | 0.75 | 0.61 | 0.56 | 7.53 | 8.24 |
| **Cortical surface areas and ICV** | 0.48 | 0.53 | 0.23 | 0.28 | 10.93 | 10.85 |

Performance metrics under 10-fold cross-validation in the training samples of males and females using features from three different modalities are displayed here. R, Pearson’s correlation; R^2^, explained variance; MAE, mean absolute error.

**Supplementary Table S9.** Feature importance of modalities.

| **Feature importance** | **Sex** | **R** | | **R^2^** | | **MAE** | |
| --- | --- | --- | --- | --- | --- | --- | --- |
|  |  | Control test sample | MDD test sample | Control test sample | MDD test sample | Control test sample | MDD test sample |
| **Full model** | M | 0.85 | 0.77 | 0.72 | 0.57 | 6.50 | 6.72 |
|  | F | 0.83 | 0.78 | 0.69 | 0.59 | 6.84 | 7.18 |
| **Subcortical volumes** | M | 0.79 | 0.71 | -6.71 | -10.03 | 41.36 | 42.52 |
|  | F | 0.78 | 0.72 | -3.41 | -4.22 | 31.34 | 30.44 |
| **Cortical thickness** | M | 0.72 | 0.63 | -40.61 | -56.45 | 98.10 | 98.79 |
|  | F | 0.69 | 0.62 | -40.21 | -48.91 | 99.98 | 98.32 |
| **Cortical surface area** | M | 0.82 | 0.73 | -3.01 | -4.74 | 29.35 | 30.09 |
|  | F | 0.81 | 0.75 | -1.65 | -2.22 | 23.81 | 23.39 |

The full model includes all orginal testdata and 77 features. The rows indicate which features (subcortical volumes [8 features] or cortical thickness [34 features] or cortical surface area [34 features]) were perturbed (values set to zero) in the test samples. Test performance is most negatively affected by the perturbation of cortical thickness features. R, Pearson correlation coefficient; R2, explained variance; MAE, mean absolute error; M, males; F, females.

**Supplementary Table S10*.*** A qualitative comparison between structure coefficients.

| **FreeSurfer Feature** | **Top 10%** | **Bottom 90%** | **Difference** |
| --- | --- | --- | --- |
| M_parahippocampal_surfavg | -0.18 | -0.41 | -0.23 |
| M_superiorparietal_surfavg | -0.12 | -0.25 | -0.14 |
| M_fusiform_surfavg | -0.25 | -0.39 | -0.14 |
| M_cuneus_surfavg | -0.03 | -0.15 | -0.12 |
| M_precentral_thickavg | -0.37 | -0.49 | -0.12 |
| M_paracentral_thickavg | -0.46 | -0.58 | -0.12 |
| M_parstriangularis_surfavg | -0.15 | -0.26 | -0.11 |
| M_precuneus_surfavg | -0.12 | -0.23 | -0.11 |
| M_superiorfrontal_thickavg | -0.58 | -0.67 | -0.09 |
| M_lingual_surfavg | -0.13 | -0.22 | -0.09 |
| M_lateraloccipital_surfavg | -0.13 | -0.22 | -0.09 |
| Mamyg | -0.19 | -0.28 | -0.09 |
| M_frontalpole_surfavg | -0.04 | -0.12 | -0.09 |
| Mvent | 0.50 | 0.41 | -0.09 |
| M_pericalcarine_surfavg | -0.02 | -0.10 | -0.08 |
| Maccumb | -0.51 | -0.59 | -0.07 |
| M_parsopercularis_thickavg | -0.51 | -0.58 | -0.07 |
| M_posteriorcingulate_surfavg | -0.10 | -0.17 | -0.07 |
| M_entorhinal_surfavg | -0.04 | -0.11 | -0.07 |
| M_bankssts_thickavg | -0.32 | -0.39 | -0.06 |
| M_precuneus_thickavg | -0.60 | -0.66 | -0.06 |
| M_cuneus_thickavg | -0.33 | -0.39 | -0.06 |
| M_superiortemporal_surfavg | -0.13 | -0.19 | -0.06 |
| M_lateralorbitofrontal_surfavg | -0.20 | -0.25 | -0.05 |
| M_parsorbitalis_surfavg | -0.12 | -0.18 | -0.05 |
| M_rostralmiddlefrontal_surfavg | -0.17 | -0.22 | -0.05 |
| M_inferiorparietal_surfavg | -0.22 | -0.27 | -0.05 |
| M_postcentral_surfavg | -0.11 | -0.16 | -0.04 |
| Mcaud | -0.35 | -0.39 | -0.04 |
| M_postcentral_thickavg | -0.41 | -0.45 | -0.04 |
| M_supramarginal_surfavg | -0.18 | -0.21 | -0.04 |
| M_transversetemporal_surfavg | -0.14 | -0.18 | -0.04 |
| M_rostralanteriorcingulate_thickavg | -0.40 | -0.43 | -0.03 |
| M_middletemporal_surfavg | -0.22 | -0.25 | -0.03 |
| M_supramarginal_thickavg | -0.49 | -0.52 | -0.03 |
| ICV | -0.10 | -0.13 | -0.03 |
| M_superiorfrontal_surfavg | -0.20 | -0.22 | -0.03 |
| Mthal | -0.50 | -0.53 | -0.03 |
| M_caudalmiddlefrontal_thickavg | -0.49 | -0.51 | -0.03 |
| M_superiorparietal_thickavg | -0.46 | -0.49 | -0.02 |
| M_caudalanteriorcingulate_surfavg | -0.14 | -0.16 | -0.02 |
| M_insula_surfavg | 0.02 | 0.00 | -0.02 |
| M_isthmuscingulate_surfavg | -0.04 | -0.05 | -0.02 |
| M_isthmuscingulate_thickavg | -0.64 | -0.66 | -0.01 |
| M_bankssts_surfavg | -0.26 | -0.28 | -0.01 |
| M_superiortemporal_thickavg | -0.49 | -0.50 | -0.01 |
| M_temporalpole_surfavg | -0.07 | -0.08 | -0.01 |
| M_inferiorparietal_thickavg | -0.54 | -0.55 | 0.00 |
| M_caudalmiddlefrontal_surfavg | -0.22 | -0.22 | 0.00 |
| Mput | -0.59 | -0.59 | 0.00 |
| M_parstriangularis_thickavg | -0.46 | -0.46 | 0.00 |
| M_rostralanteriorcingulate_surfavg | -0.21 | -0.20 | 0.00 |
| M_caudalanteriorcingulate_thickavg | -0.39 | -0.39 | 0.01 |
| M_pericalcarine_thickavg | -0.27 | -0.27 | 0.01 |
| M_medialorbitofrontal_surfavg | -0.14 | -0.13 | 0.01 |
| M_parsopercularis_surfavg | -0.28 | -0.26 | 0.01 |
| M_posteriorcingulate_thickavg | -0.66 | -0.65 | 0.01 |
| Mpal | -0.09 | -0.08 | 0.01 |
| M_rostralmiddlefrontal_thickavg | -0.41 | -0.39 | 0.02 |
| M_frontalpole_thickavg | -0.35 | -0.33 | 0.02 |
| M_inferiortemporal_surfavg | -0.25 | -0.22 | 0.02 |
| M_transversetemporal_thickavg | -0.49 | -0.46 | 0.03 |
| M_lingual_thickavg | -0.43 | -0.39 | 0.03 |
| Mhippo | -0.42 | -0.39 | 0.04 |
| M_parsorbitalis_thickavg | -0.40 | -0.36 | 0.04 |
| M_paracentral_surfavg | -0.05 | -0.01 | 0.04 |
| M_insula_thickavg | -0.55 | -0.50 | 0.05 |
| M_medialorbitofrontal_thickavg | -0.34 | -0.28 | 0.05 |
| M_precentral_surfavg | -0.13 | -0.07 | 0.06 |
| M_temporalpole_thickavg | -0.01 | 0.05 | 0.06 |
| M_lateraloccipital_thickavg | -0.39 | -0.29 | 0.10 |
| M_parahippocampal_thickavg | -0.21 | -0.11 | 0.10 |
| M_middletemporal_thickavg | -0.43 | -0.31 | 0.12 |
| M_lateralorbitofrontal_thickavg | -0.34 | -0.22 | 0.12 |
| M_fusiform_thickavg | -0.40 | -0.26 | 0.15 |
| M_inferiortemporal_thickavg | -0.30 | -0.13 | 0.17 |
| M_entorhinal_thickavg | -0.02 | 0.18 | 0.20 |

The top 10% column indicates major depressive disorder patients with the highest decile of brain-PAD values. The difference is sorted from smallest to largest and reflects the difference compared to the bottom 90% of brain-PAD values.
