# Supplementary material for: Brain aging in major depressive disorder: results from the ENIGMA major depressive disorder working group
Source: Mol Psychiatry. 2020 May 18;26(9):5124–39. doi: 10.1038/s41380-020-0754-0 (PMC8589647; doi:10.1038/s41380-020-0754-0)
Supplement: Supplementary file 2 — Supplementary Materials [file 41380_2020_754_MOESM2_ESM.docx]

**Brain aging in Major Depressive Disorder - Supplementary Material**

**Image exclusion criteria**

A neuroimaging expert at each scanning site inspected each image segmentation by overlaying the segmentation label of each structure on the T1-weighted brain scan. Additionally, study-wide statistics were collected (means and standard deviations) as well as histogram plots to identify non-normally distributed data and major outliers. Samples were excluded if its FreeSurfer feature was >2.698 standard deviations away from the global mean. If a sample was marked as a statistical outlier, the individual site was asked to re-inspect the subject’s segmentation in order to verify that it was properly segmented. If a sample was a statistical outlier, yet properly segmented, it was kept in the dataset. Otherwise, the sample was removed.

**Quality checking and sample exclusion criteria**

The initial dataset included 35 scanning sites with an age range of 7-89 years old based on N=8,369 samples. However, due to scarcity of samples around upper age boundary and to develop an adult model in the context of aging, we excluded those below 18 years and above 75 years old. Subsequently, the chronological age variable was floored, as some sites included one or two decimals in their age variable, while others did not. We checked individual FreeSurfer features for missings and excluded participant samples with >10% missing data, suggestive of poor reliability. The above criteria led to an exclusion of N=713 participants, resulting in the total sample of N=7,656.

The total sample of (N=7,656) was partitioned into datasets of controls and major depressive disorder (MDD) patients, separately for males and females, including N=2,158 male controls and N=1,139 male MDD patients, and N=2,532 female controls and N=1,827 female MDD patients.

**Data partitioning**

We divided healthy controls from each of the scanning sites into separate training (subset to train the model) and test samples (subset to test the trained model) using a balanced split-half approach. The 50:50 data partitioning was performed following random sampling within each of these scanning sites while preserving the chronological age distribution between training and test data using the createDataPartition function from the “*caret*” package in R. Whereas multiple approaches to partitioning datasets intro training and test samples were possible, we would like to explain our motivation for the specific approach used. By dividing the control subjects from each scanning site into equal parts of training and test data, we optimally maximize: a) the chronological age distribution and variety of scanning sites in our training set to increase generalizability, and b) the statistical power and sample size of MDD patients for subsequent statistical analyses in the test sets to answer our main research question. To clarify, not all scanning sites cover the wide age range (18-75 years of age) and chronological age and scanning site were therefore correlated.

Following an alternative data partition approach, we could train our model on control subjects from scanning sites (e.g. A, B, C) to test on a held-out set of other scanning sites (e.g. X, Y, Z). However, with that approach, we would need to exclude the corresponding MDD patients from those sites (A, B, C) from subsequent statistical analyses performed on the test data (i.e. controls vs. MDD patients). This is because the model would be trained on controls from the same scanning sites as those patients (both A, B, C), whereas the remaining patients and controls would be from completely independent scanning sites (X, Y, Z). If MDD patients from those scanning sites would be included, it would introduce a bias of learned patterns from the scanning sites for a part of the MDD patients (A, B, C) but not for the other part of MDD patients or any of the healthy controls (X, Y, Z). As a wider variety of scanners also improves the subsequent generalization to independent unseen data, we have opted for the data partition approach reported in the main manuscript. This is further supported by the generalization of our multisite models to the ENIGMA Bipolar Disorder working group dataset collected from 23 independent scanning sites.

Scanning sites with less than 20 control samples were excluded from the data partitioning, ensuring that training and test datasets both included at least 10 samples. Corresponding MDD patients from those scanning sites were also excluded. Thus, 16 scanning sites remained in the male sample (N=279 participants were excluded), compared to 22 scanning sites in the female sample (N=97 participants were excluded). The final training sample consisted of N=952 male controls and N=1,236 female controls. The final test samples consisted of N=927 male controls and N=986 depressed males and N=1,199 female controls and N=1,689 female depressed patients.

**Brain age prediction framework**

**Alternative machines/kernels**

To explore the effect of different machines and kernels, we repeated the 10-fold cross-validation training using Support Vector Regression (SVR) and Random Forest Regression (RFR) in comparison to the Ridge Regression. To model non-linear multivariate patterns, we also explored radial basis function (RBF) kernels, as compared to linear kernel methods. Important to mention here is that all machine learning algorithms showed similar performances (**Supplementary Table S6**). Given the aim to make our model publicly available, we opted for the Ridge Regression emphasizing its deployability and shareability. In contrast to the RBF kernels, Ridge Regression allows for sharing model weights at the feature level for making predictions in new independent test samples, without sharing any actual data points or support vectors from the training data. This ensures that no individual level data is shared.

**The correlation between brain-PAD and chronological age**

We observed a significant correlation between brain-PAD and chronological age (**Supplementary Figure S1A and S1B; Supplementary Table S7**), which is a known phenomenon in any brain age prediction framework. Since age is the variable being predicted, the model is fitted to minimize the error around predicted age and not the error around observed age. This commonly leads to young people being systematically predicted to be older and old people to be systematically predicted younger. Currently, three excellent papers describe this “regression dilution” phenomenon in brain age prediction models and propose solutions to statistically deal with this bias (Le et al., 2018; Liang, Zhang and Niu, 2018; Smith et al., 2019).[^1–3^](https://paperpile.com/c/6D0aK4/lbuOv+lmsOL+jGRiv) We would like to emphasize that the aim and scope of our paper were to test whether MDD patients show higher brain-PAD than controls. To this aim we have applied a statistical method in which we account for this bias (further described below) to answer this research question, thus our conclusions are not affected by the systematic effect of the regression dilution.


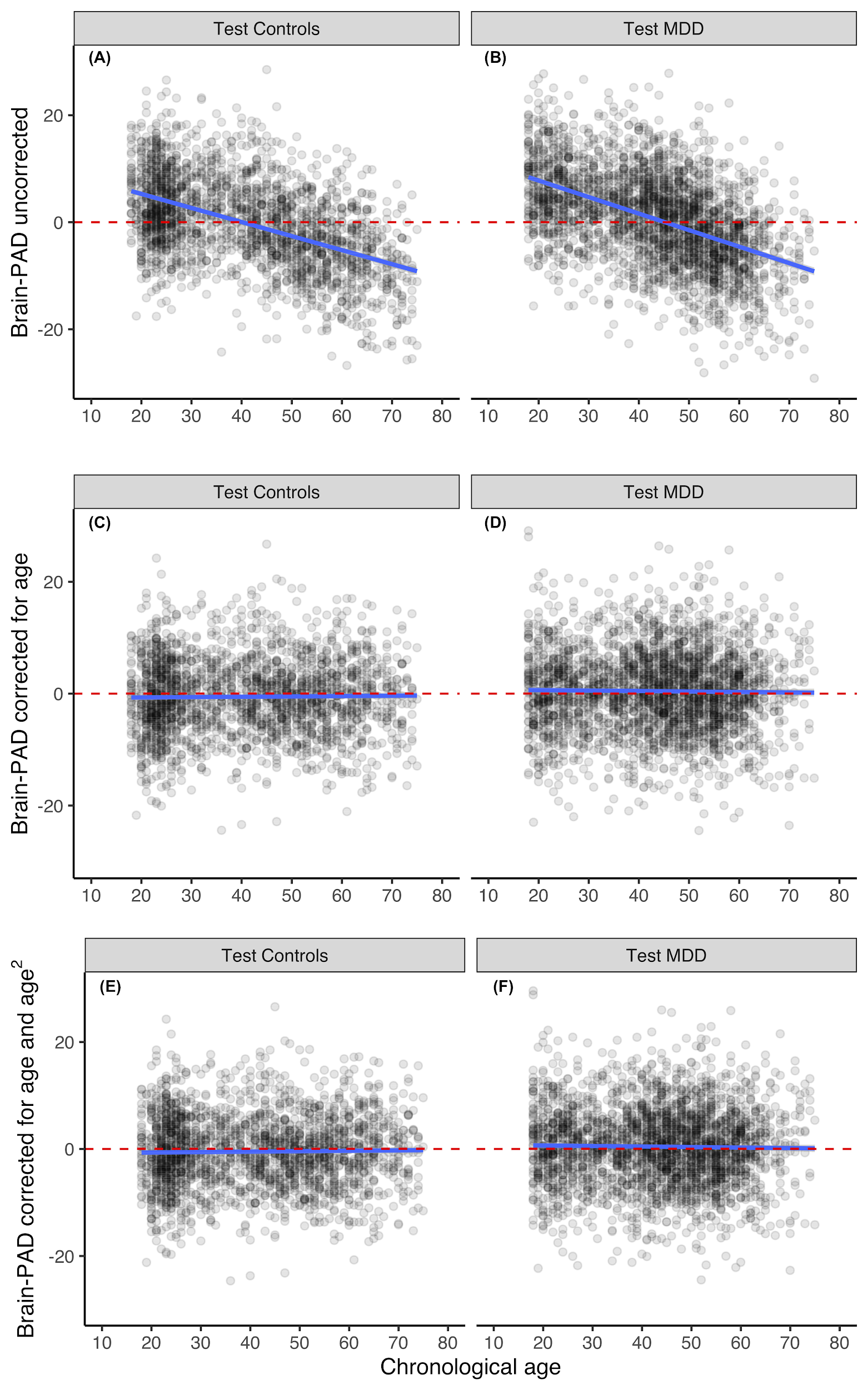


**Supplementary Figure S1.** Brain-PAD was significantly negatively correlated to chronological age (overall r=-0.48, p<0.0001) in both controls **(A)** and MDD patients **(B)**. After linear correction brain-PAD was not significantly correlated to chronological age (r=-2.59E-15, p=1) in neither controls **(C)** nor MDD patients **(D)**. Although difficult to visually detect, additional nonlinear correction for age^2^ resulted in a better model fit in both controls **(E)** and MDD patients **(F)** compared to correction for linear age effects only (χ_(2)_=9.73, p<0.002).

**Correcting the linear and nonlinear age dependence of brain-PAD**

Regression dilution, described above, creates a dependence between chronological age and predicted age. To control for this effect, we have included chronological age as a covariate in subsequent analyses (**Supplementary Figure S1C and S1D)** as proposed in Le et al. (2018).[^1^](https://paperpile.com/c/6D0aK4/lbuOv) This removes all linear age dependence of our outcome variable. However, one can assume that not all aging effects are perfectly linear. More specifically, the morphology of the brain follows nonlinear trajectories in the young and adolescent,[^4^](https://paperpile.com/c/6D0aK4/SesX7) but we may potentially also expect an acceleration in older ages, specifically with respect to (mental) illness.[^2,5^](https://paperpile.com/c/6D0aK4/lmsOL+bC3E1) As Smith and colleagues (2019) suggest the bias in brain age estimation and the nonlinear dependence can be adjusted for. To statistically correct for the nonlinear age effects on the brain-PAD metric, we included quadratic age terms in our models to test group differences in our test samples (**Supplementary Figure S1E and S1F**). Important to note, we formally tested the goodness of fit of models including age only, age and age^2^, and age, age^2^ and age^3^ as covariates, and found that the model including age and quadratic age terms statistically showed the best model fit.

**Statistical analyses of case-control comparison of brain-PAD**

By including age and nonlinear age effects as covariates in all statistical models, we statistically adjusted for the systematic age bias but also for any other potentially confounding effects of age in our analyses. Moreover, we demonstrate that there were no significant interactions with age or age^2^ and MDD status. Thus, the residual age effects in the brain-PAD metric did not influence our main finding with regard to case-control differences.

**Alternative feature selection: single modality trained models**

We explored if certain selections of features would optimally explain the age variance in the data. Therefore, we built three separate models with the aim to reduce feature space by including only single modalities (only cortical thickness vs. cortical surface area vs. subcortical volume features). The cortical thickness and intracranial volume (ICV) and subcortical volume and ICV showed reasonable performance accuracy (MAE=7.53-8.95), but a model only trained on surface area features and ICV performed the worst (MAE=10.9 years in both males and females). Combining all 77 features and maximizing the feature set resulted in the most superior performance accuracy (MAE=6.3-6.6), **Supplementary Table S8**. Please note that we used the following formula implemented in the *caret* package in *R* to calculate R^2^:

*R^2^ = 1-\frac{∑ (y_i - \hat{y}_i)^2^}{∑ (y_i - \bar{y}_i)^2^}*

This formula also allows negative expressions of R^2^, indicating that despite the high correlation between chronological age and predicted brain age, the data are not well-explained by the model.

**Feature importance of modalities**

To get a sense of how important a feature is for prediction, we systematically set certain features to zero in all test samples. Overall, cortical thickness features seem to be important for obtaining good predictions, as the MAE increases to >98 years when perturbed (**Supplementary Table S9**). The MAEs were equally affected in control and MDD patients, suggesting that the features important for making accurate brain age predictions were similar across groups.

**Qualitative comparison**

To qualitatively compare the MDD patients with the highest decile of brain-PAD to the bottom 90%, we have provided structure coefficients between predicted brain age and all features. In short, the parahippocampal surface area showed the largest differential structure coefficient, with a diminished reduction in the top 10% compared to the bottom 90%. The parahippocampal region is an essential input region to the hippocampus, a structure that is commonly implicated in MDD. The reported increased parahippocampal surface area in MDD by Qui and colleagues (2014) is in line with the current observation, however, this surface area difference could not be replicated by Peng et al. (2015).[^6,7^](https://paperpile.com/c/6D0aK4/c45Ir+2ExXo) Taken together, we observed that particularly thickness features were more negatively associated with predicted brain age in the top 10% highest brain-PAD patients compared to the bottom 90%, while this was vice versa for surface area features (**Supplementary Table S10**).

**Generalizability to independent test samples from the ENIGMA MDD working group**

The brain age prediction model generalized well to unseen samples. The overall correlations between predicted brain age and chronological age in the out-of-sample test controls from the ENIGMA MDD working group were r=0.85, P<0.001; R^2^=0.72 for males and r=0.83,p<0.001; R^2^=0.69 for females. Similarly, the performances in the MDD test samples were r=0.77, p<0.001; R^2^=0.57 for males, and r=0.78, p<0.001; R^2^=0.59) for females. Of note here is that prediction errors were similar, but not equal between sites and age groups (**Supplementary Figures S2-5**). More specifically, the mean absolute error (MAE) was highest in the oldest age group (68-75 years old, mean 10.25 [6.59]), although this group was relatively small (N=166 out of N=4,801). Brain predicted age difference (brain-PAD) was significantly negatively associated with chronological age (overall r=-0.48, p<0.0001).

**Supplementary Figure S2. Mean absolute error (MAE) and brain predicted age difference (brain-PAD) across scanning site and age group for the male control test samples.** Top row figures illustrate scanning sites on the x-axis. Prediction errors were examined across 16 different scanning sites and six different age groups of ten-year bins.

**Supplementary Figure S3. Mean absolute error (MAE) and brain predicted age difference (brain-PAD) across scanning site and age group for the male major depression disorder (MDD) test samples.** Top row figures illustrate scanning sites on the x-axis. Prediction errors were examined across 16 different scanning sites and six different age groups of ten-year bins.

**Supplementary Figure S4. Mean absolute error (MAE) and brain predicted age difference (brain-PAD) across scanning site and age group for the female control test samples.** Top row figures illustrate scanning sites on the x-axis. Prediction errors were examined across 22 different scanning sites and six different age groups of ten-year bins.

**Supplementary Figure S5. Mean absolute error (MAE) and brain predicted age difference (brain-PAD) across scanning site and age group for the female major depression disorder (MDD) test samples.** Top row figures illustrate scanning sites on the x-axis. Prediction errors were examined across 22 different scanning sites and six different age groups of ten-year bins.

**Generalizability to completely independent healthy controls from the ENIGMA BD working group**

The brain age prediction models generalized well to healthy controls from completely independent samples (i.e. independent scanning sites) from the ENIGMA Bipolar Disorder (BD) working group (**Supplementary Figures S6-7**). The MAE was 7.49 (5.89) years in males and 7.26 (SD 5.63) in females, slightly higher than the MAE in the test samples of the ENIGMA MDD working group. The overall correlations between predicted brain age and chronological age in the out-of-sample controls were r=0.71, p<0.001; R^2^=0.45 for males and r=0.72, p<0.001; R^2^=0.48 for females.

**Supplementary Figure S6. Mean absolute error (MAE) and brain predicted age difference (brain-PAD) across scanning site and age group for the male control test sample from the ENIGMA Bipolar Disorder (BD) working group.** Top row figures illustrate scanning sites on the x-axis. Prediction errors were examined across 23 different scanning sites and six different age groups of ten-year bins.

**Supplementary Figure S7. Mean absolute error (MAE) and brain predicted age difference (brain-PAD) across scanning site and age group for the female control test sample from the ENIGMA Bipolar Disorder (BD) working group.** Top row figures illustrate scanning sites on the x-axis. Prediction errors were examined across 23 different scanning sites and six different age groups of ten-year bins.

**ENIGMA MDD Brain Age Model publicly available**

**Public availability of the ENIGMA brain age model**

FreeSurfer is an automated and widely used software tool (<http://surfer.nmr.mgh.harvard.edu/>). Thus, our brain age algorithm can be easily applied to independent data, promoting validation and replication across different samples worldwide needed to mature modeling efforts, contributing to the development of canonical brain age models. To this aim, our FreeSurfer-based brain age model has been made publicly available at [https://www.photon-ai.com](https://www.photon-ai.com/brainage)/enigma_brainage. Detailed instructions and guidelines for its use will be made available on the webpage. It is, however, important to note that prediction errors were higher in older age groups (>60 years old) and brain-PAD was significantly negatively associated with chronological age (r=-0.53 males, r=-0.48 females, both p’s<0.0001), with the latter being a known feature of the brain-PAD metric.[^1^](https://paperpile.com/c/6D0aK4/lbuOv) Thus, caution is warranted when applying our model to data from older participants (>60 years). We recommend to: a) only use our models to samples with an upper age limit of 60 years, and b) always include residual chronological age effects as covariates in the analyses.

**Feature importance: structure coefficients**

All features, except the mean lateral ventricle volume, and entorhinal and temporal pole thickness showed a negative correlation with predicted brain age, and are visualized in **Figure 4** in the main manuscript. Widespread negative correlations with average cortical thickness and surface area were observed, although thickness features resulted in stronger negative correlations (mean Pearson r [SD]: -0.44 [0.21]) than surface area features (-0.17 [0.08]). On average, subcortical volumes were slightly less negatively correlated to predicted brain age as thickness features (-0.34 [0.34]). We also visualized these associations separately for controls and MDD patients, but findings were similar and suggest comparable structure coefficients in both groups (**Supplementary Figure S8**).


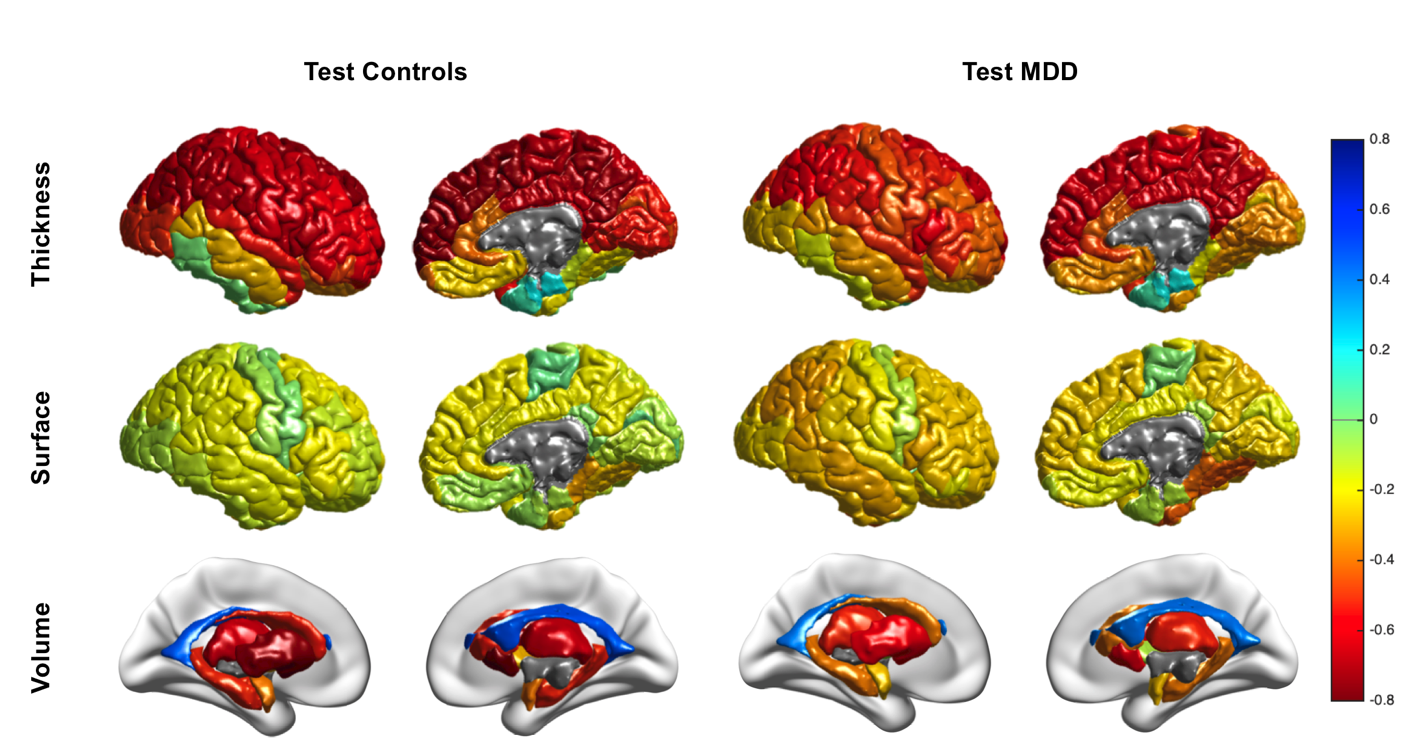


**Supplementary Figure S8. Structure coefficients of predicted brain age and FreeSurfer features between control and major depressive disorder (MDD) groups.** Bivariate correlations are shown for illustrative purposes and to show the similarity of patterns between controls and MDD patients. The figure shows Pearson correlations between predicted brain age and cortical thickness features (*top row*), cortical surface areas (*middle row*), and subcortical volumes (*bottom row*). The negative correlation with intracranial volume (ICV) was excluded from this figure for display purposes.

**References**

1 [Le TT, Kuplicki RT, McKinney BA, Yeh H-W, Thompson WK, Paulus MP *et al.* A Nonlinear Simulation Framework Supports Adjusting for Age When Analyzing BrainAGE. *Front Aging Neurosci* 2018; **10**: 317.](http://paperpile.com/b/6D0aK4/lbuOv)

2 [Smith SM, Vidaurre D, Alfaro-Almagro F, Nichols TE, Miller KL. Estimation of Brain Age Delta from Brain Imaging. 2019. doi:](http://paperpile.com/b/6D0aK4/lmsOL)[10.1101/560151](http://dx.doi.org/10.1101/560151)[.](http://paperpile.com/b/6D0aK4/lmsOL)

3 [Liang H, Zhang F, Niu X. Investigating systematic bias in brain age estimation with application to post‐traumatic stress disorders. *Hum Brain Mapp* 2019; **10**: 1.](http://paperpile.com/b/6D0aK4/jGRiv)

4 [Wierenga LM, Langen M, Oranje B, Durston S. Unique developmental trajectories of cortical thickness and surface area. *Neuroimage* 2014; **87**: 120–126.](http://paperpile.com/b/6D0aK4/SesX7)

5 [Fjell AM, Westlye LT, Grydeland H, Amlien I, Espeseth T, Reinvang I *et al.* Critical ages in the life course of the adult brain: nonlinear subcortical aging. *Neurobiol Aging* 2013; **34**: 2239–2247.](http://paperpile.com/b/6D0aK4/bC3E1)

6 [Qiu L, Lui S, Kuang W, Huang X, Li J, Li J *et al.* Regional increases of cortical thickness in untreated, first-episode major depressive disorder. *Transl Psychiatry* 2014; **4**: e378.](http://paperpile.com/b/6D0aK4/c45Ir)

7 [Peng D, Shi F, Li G, Fralick D, Shen T, Qiu M *et al.* Surface vulnerability of cerebral cortex to major depressive disorder. *PLoS One* 2015; **10**: e0120704.](http://paperpile.com/b/6D0aK4/2ExXo)
